# High Electrical Conductivity Induced by Surface Confinement Effect in Heterostructured Multifunctional Nanofiber Composite Films for Low‐Reflection Electromagnetic Interference Shielding

**DOI:** 10.1002/advs.202510386

**Published:** 2025-07-29

**Authors:** Dechang Tao, Xin Wen, Shuai Ma, Can Chen, Jianing Guo, Wenwen Wang, Kun Yan, Chenguang Yang, Dong Wang

**Affiliations:** ^1^ Key Laboratory of Textile Fiber and Products (Wuhan Textile University) Ministry of Education Wuhan Textile University Wuhan 430200 China; ^2^ College of Chemistry Chemical Engineering and Biotechnology Donghua University Shanghai 201620 China; ^3^ Department of Cardiovascular Surgery Zhongnan Hospital of Wuhan University Wuhan 430060 China

**Keywords:** AgNW/MXene, electromagnetic interference, flexible sensing, low reflection, surface confinement effect, thermal management

## Abstract

Electromagnetic interference (EMI) shielding films with low‐reflection characteristics are ideal for blocking electromagnetic(EM) radiation and pollution. Poly(vinyl alcohol‐co‐ethylene) (PVA‐co‐PE) nanofibers, with high specific surface area and abundant active groups, can be compounded with various conductive materials to prepare EMI shielding materials with low‐reflection features. However, the large‐scale industrial application of such shielding materials with multifunctionality remains rarely explored. 1D silver nanowires (AgNW) and 2D MXene are tend to form 3D network structure, and their magnetoelectric synergistic effect can significantly enhance the energy loss of EM waves. In this study, heterostructured PVA‐co‐PE/AgNW/MXene (PAM_x_) composite films are prepared via a two‐step spray coating and solvent evaporation method. This method achieves the denseness of AgNW/MXene on the film surface through the surface confinement effect, significantly improving the utilization rate of conductive media. A 5.4 wt.% AgNW/MXene content, the EMI shielding efficiency (EMI SE), normalized SE (EMI SE/t), and absolute SE (SSE/t) are 60.2 dB, 5452.9 dB·cm^−1^ and 36352.7 dB·cm^2^·g^−1^ in the ultra‐wide frequency of 8–20 GHz, along with an extremely low reflection coefficient (R = 0.13). The straightforward preparation process makes it highly suitable for industrial‐scale production, showing significant potential in aerospace, flexible wearables fields.

## Graphical abstract

Spray‐coated PVA‐co‐PE/AgNW/MXene films achieve 60.2 dB EMI shielding (8–20 GHz) with low reflection (R = 0.13) at 5.4 wt.% loading. The 3D conductive network enhances absorption, while the film offers high strength (18.33 MPa), Joule heating, photothermal conversion, flexibility, and flame resistance. Stable after 10 000 compressions, this scalable method suits industrial use in aerospace, wearables, and thermal management.

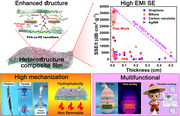

## 1 Introduction

With the rapid advancement and pervasive integration of wireless technologies in mobile communications, military systems, and smart wearable electronics,^[^ ^1^, ^2^, ^3^ ^]^ human life has experienced unprecedented convenience. However, this progress has concurrently exacerbated electromagnetic (EM) pollution and radiation challenges.^[^ ^4^, ^5^, ^6^, ^7^ ^]^ These EM waves not only disrupt the normal operation of precision instruments and electronic systems but also pose significant risks to human health.^[^ ^8^, ^9^, ^10^ ^]^ For instance, EM waves, when excessively absorbed by human tissues, are converted into thermal energy, leading to elevated body temperatures and potentially acute or chronic physiological damage.^[^ ^11^, ^12^, ^13^ ^]^ Thus, the development of high‐performance electromagnetic interference (EMI) shielding materials is urgently needed to address the escalating EM pollution crisis.^[^ ^14^, ^15^ ^]^ Traditional EMI shielding materials rely primarily on reflection‐dominated mechanisms, which inadvertently induce secondary EM wave pollution.^[^ ^6^, ^11^, ^16^ ^]^ Consequently, next‐generation EMI shielding materials must achieve the dual objectives of maximizing EM shielding effectiveness (EMI SE) and minimizing reflectivity.^[^ ^17^, ^18^, ^19^ ^]^

In addition, heat accumulation during electronic device operation requires materials with exceptional thermal management capabilities,^[^ ^20^, ^21^ ^]^ including rapid heat dissipation,^[^ ^22^ ^]^ photothermal conversion,^[^ ^6^, ^23^ ^]^ Joule heating performance,^[^ ^4^, ^12^, ^24^ ^]^ and resilience to extreme environments.^[^ ^25^ ^]^ The proliferation of smart wearable electronics and intelligent textiles requires materials that are lightweight, ultrathin, mechanically robust, and endowed with strain‐sensing functionalities for human motion monitoring.^[^ ^11^, ^26^, ^27^ ^]^ Nevertheless, existing materials frequently face challenges in integrating efficient low‐reflection EMI shielding, superior mechanical properties, advanced thermal management, and strain sensing, which significantly restricts their real‐world applications.^[^ ^28^, ^29^ ^]^ Consequently, there is an urgent need to engineer novel composite materials with specialized architectures to overcome these performance bottlenecks.

MXene, has garnered extensive research attention in EMI shielding applications owing to its exceptional electrical conductivity and high specific surface area.^[^ ^30^, ^31^, ^32^, ^33^, ^34^, ^35^ ^]^ However, pristine MXene films inherently exhibit pronounced electromagnetic wave (EMW) reflectivity, which may lead to secondary EM pollution.^[^ ^36^, ^37^, ^38^ ^]^ Therefore, MXene is often composited with other substrates to fabricate efficient EMI shielding films.^[^ ^39^, ^40^, ^41^, ^42^ ^]^ Wang et al.^[^ ^43^ ^]^ developed biodegradable MXene Ti_3_C_2_T_x_/gelatin/sodium lignosulfonate (MXene/GEL/SL) films through a facile one‐pot synthesis. For a composite film with 50 wt.% MXene loading, they achieved a total shielding efficiency (SE_T_) of 47 dB in the X‐band, with an absorption‐dominated shielding efficiency (SE_A_) of ≈36 dB. Similarly, Li et al.^[^ ^44^ ^]^ employed electrospinning, vacuum filtration, and hot‐pressing techniques to fabricate EVA/MXene/EVA composite films, which exhibited an EMI SE of 49.6 dB in the 8.2–12.4 GHz frequency range with 100 mg of MXene.

Although the aforementioned approaches enable effective EMI shielding, they rely predominantly on reflection‐dominated mechanisms and fail to meet the critical requirement of low reflectivity. Moreover, the incorporation of high‐conductivity fillers often compromises the mechanical robustness and flexibility of the films.^[^ ^45^, ^46^, ^47^ ^]^ Consequently, the development of multifunctional composite films with superior mechanical properties and efficient low‐reflection EMI shielding necessitates reducing the filler loading and devising innovative fabrication strategies. Considering that the surface confinement effect can restrict conductive media to specific interfacial regions, thereby significantly improving their utilization efficiency, and that hierarchical structures within materials can enhance the reflection and refraction of EM waves,^[^ ^18^ ^]^ constructing specialized surface structures in composite films via the surface confinement effect is a viable strategy to boost wave absorption. This approach promotes in‐film wave propagation, mitigates secondary pollution, and prolongs transmission paths to enhance wave dissipation.^[^ ^6^, ^7^, ^48^ ^]^ Yuan et al.^[^ ^49^ ^]^ fabricated MXene‐carbon fiber paper (CPML‐n) EMI shielding composites using a hybrid wet papermaking and spray impregnation method. With a low MXene loading of 0.4 wt.% and 7 spray layers, the composites achieved a high EMI SE of 78.23 dB, with an SE_A_ of 70 dB and a significantly reduced reflection coefficient. In another study, Yang et al.^[^ ^13^ ^]^ imprinted filter material architectures onto film surfaces during vacuum filtration, leveraging twill‐structured nylon 6 fabrics to engineer tailored surface topologies. Poly(vinyl alcohol‐co‐ethylene) (PVA‐co‐PE) nanofiber‐based films, known for their biocompatibility and flexibility, are ideal substrates for smart wearables and have been widely used in flexible multifunctional composites.^[^ ^50^, ^51^, ^52^ ^]^

Building on the above findings, this study presents the fabrication of heterostructured nanofiber composite films (PVA‐co‐PE/AgNW/MXene, PAM_x_) using PVA‐co‐PE nanofibers and a polypropylene (PP) nonwoven fabric as the functional matrix and structural support, respectively, with 1D AgNW and 2D MXene serving as the conductive components, which are more prone to form a 3D network structure. The films were fabricated via a two‐step spray coating process, followed by solvent evaporation, glutaraldehyde‐induced chemical crosslinking, and interfacial hydrogen bonding. Initially, glutaraldehyde crosslinking improved inter‐fiber adhesion within the PVA‐co‐PE network. Subsequently, the surface confinement effect enables the dense distribution of AgNW/MXene on the film surface. AgNW acts as a “bridge” to closely connect MXene and nanofibers through abundant hydrogen bonds, facilitating robust interfacial interactions and assembling into a stable 3D network structure. Their magnetoelectric synergistic effect can significantly enhance the energy loss of EM waves. The hierarchical reinforcement of interfacial bonding and excellent intrinsic conductivity of the fillers enabled the PAM_x_ films to achieve high electrical conductivity at low filler concentrations. Consequently, the heterostructured PAM_x_ films exhibited superior mechanical robustness and efficient low‐reflection EMI shielding. The influence of the AgNW/MXene loading on the mechanical strength and EMI shielding effectiveness was systematically investigated. Compared to conventional hybrid configurations, the heterostructured design significantly enhanced the EMW absorption and overall EMI shielding performance. Environmental stability assessments demonstrated that the PAM_x_ films retained their structural integrity and functional performance even under extreme conditions. In addition, they exhibited outstanding electrothermal and photothermal conversion capabilities, including low‐voltage Joule heating and rapid thermal responsiveness. The films also displayed flexible sensing functionality for real‐time human motion detection, along with hydrophobicity and flame‐retardant properties. This work introduces a structurally engineered, multifunctional composite film with low‐reflection EMI shielding, offering strong potential for integration into next‐generation smart wearable electronics, aerospace systems, and healthcare monitoring applications.

## 2 Results and Discussion

### 2.1 Preparation of Heterostructured PAM_x_ Composite Films

**Figure 1:**
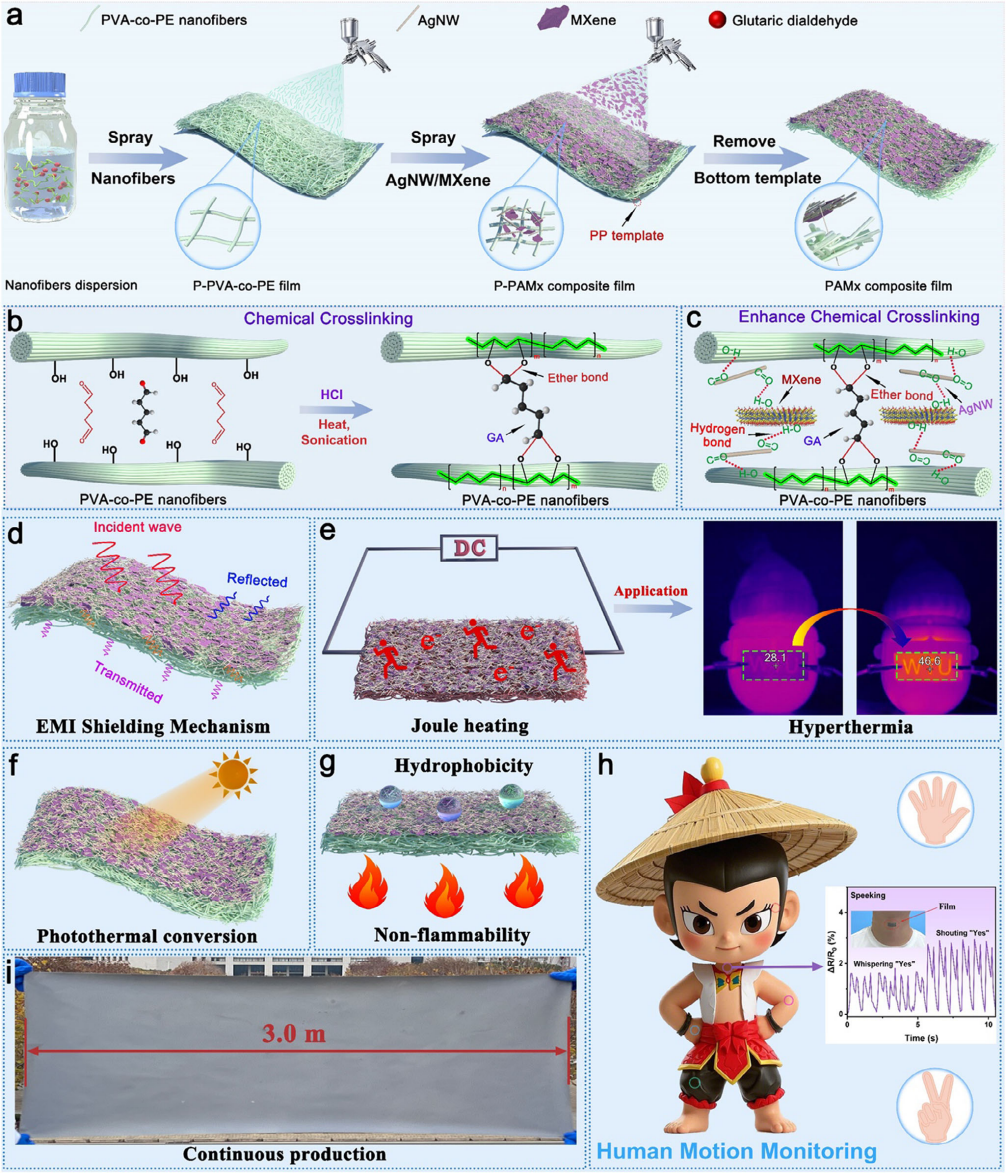
a) Schematic of the fabrication process of PAM_x_ composite films. b) Chemical crosslinking between PVA‐co‐PE nanofibers and glutaraldehyde. c) Hydrogen bonding between PVA‐co‐PE and AgNW/MXene. Multifunctionalization of PAM_x_. d) EMI shielding, e) Joule heating, f) Photothermal conversion, g) Hydrophobicity, h) Sensing performance (The cartoon character was generated by Jimeng AI), i) Industrial‐scale production.

**Figure** **1**a shows a schematic of the two‐step spray‐coating method combined with solvent evaporation for preparing PAM_x_. First, using the nonwoven PP fabric as the spraying substrate, the crosslinked PVA‐co‐PE nanofiber suspension was spray‐deposited onto a PP carrier. After solvent evaporation, a predetermined amount of AgNW/MXene was sprayed onto the P‐PVA‐co‐PE films. Once the spraying was complete and the solvent had evaporated, the PP carrier was peeled off to obtain heterostructured PAM_x_. Figure 1b shows a schematic of the chemical crosslinking between glutaraldehyde and PVA‐co‐PE in a hydrochloric acid environment, enabling the construction of an interfacial reinforcement network. Figure 1c illustrates the hydrogen‐bond‐enhanced interactions between PVA‐co‐PE, AgNW, and MXene, which strengthen the interfacial forces between AgNW/MXene and the nanofiber film, thereby improving the performance stability of the multifunctional composite film. AgNW exhibit a high electrical conductivity and aspect ratio, making them more likely to form a complete 3D conductive network with 2D MXene. Additionally, the excellent conductivity and extremely high energy conversion efficiency of AgNW/MXene, which can generate substantial heat under low‐voltage driving, endow PAM_x_ with superior EMI shielding performance (Figure 1d), ideal Joule heating properties (Figure 1e), excellent photothermal conversion performance (Figure 1f), hydrophobic and nonflammable characteristics (Figure 1g), and potential flexible sensing performance (Figure 1h). The excellent dispersibility of AgNW/MXene in solvents such as water, ethanol, and isopropanol enables industrial‐scale production of PAM_x_ (Figure 1i).

### 2.2 Microstructure Analysis

**Figure 2:**
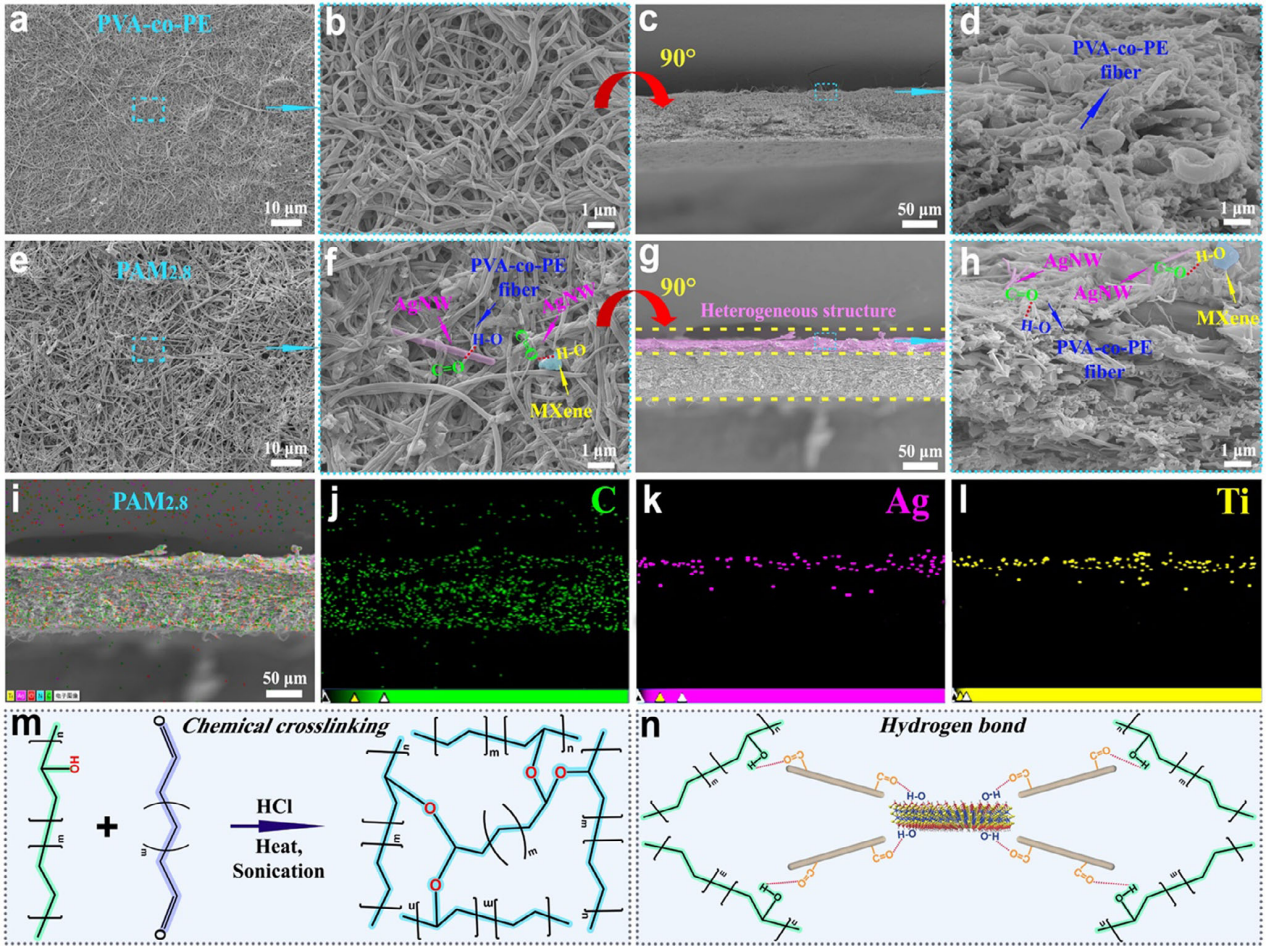
Low and high magnification SEM images of the PVA‐co‐PE nanofiber film. a,b) Surface morphology, c,d) Cross‐sectional morphology. Low and high magnification SEM images of PAM_2.8_. e,f) Surface morphology, g‐h) Cross‐sectional morphology. Cross‐sectional EDS elemental mapping of PAM_2.8_, i) Morphology, j) C, k) Ag, and l) Ti. m) Glutaraldehyde chemical crosslinking reaction. n) Hydrogen bonding interactions among nanofibers, AgNW, and MXene.

**Figure** **2** shows the surface, cross‐section, elemental distribution, and chemical interaction diagrams of the PVA‐co‐PE nanofiber films and PAM_x_. Figure 2a–d shows the surface and cross‐sectional SEM images of the PVA‐co‐PE nanofiber film. Compared with those on the non‐AgNW/MXene surface of the glutaraldehyde‐crosslinked PAM_2.8_ (Figure S4a‐a″, Supporting Information), the interfacial forces between the PVA‐co‐PE nanofibers in the crosslinked composite film were significantly enhanced, exhibiting a denser structure. The chemical crosslinking mechanism is shown in Figure 2m. As shown in Figure 2e,f, and Figure S4 (Supporting Information), with an increase in AgNW/MXene content, AgNW/MXene was distributed uniformly on the surface of PAM_x_, and the interaction forces with the nanofibers were stronger, forming a stable conductive network structure. This indicates that hydrogen bonds may have been formed between the AgNW, MXene, and PVA‐co‐PE nanofibers (Figure 2n). Figure 2g,h show the cross‐sectional SEM images of PAM_2.8_. With an increase in the AgNW/MXene content, the density of PAM_x_ increased, leading to an increase in its thickness (Figure S5, Supporting Information). Figure 2i–l presents the distribution of different elements and the energy‐dispersive spectroscopy (EDS) pattern of PAM_2.8_ (Figure S6, Supporting Information). Ag and Ti were distributed uniformly on a single surface of the composite film, forming a distinct heterostructure. In summary, the heterostructured PAM_x_ prepared in this study exhibited a stable conductive network structure, laying the foundation for efficient EMI shielding.

### 2.3 Structural Analysis

**Figure 3:**
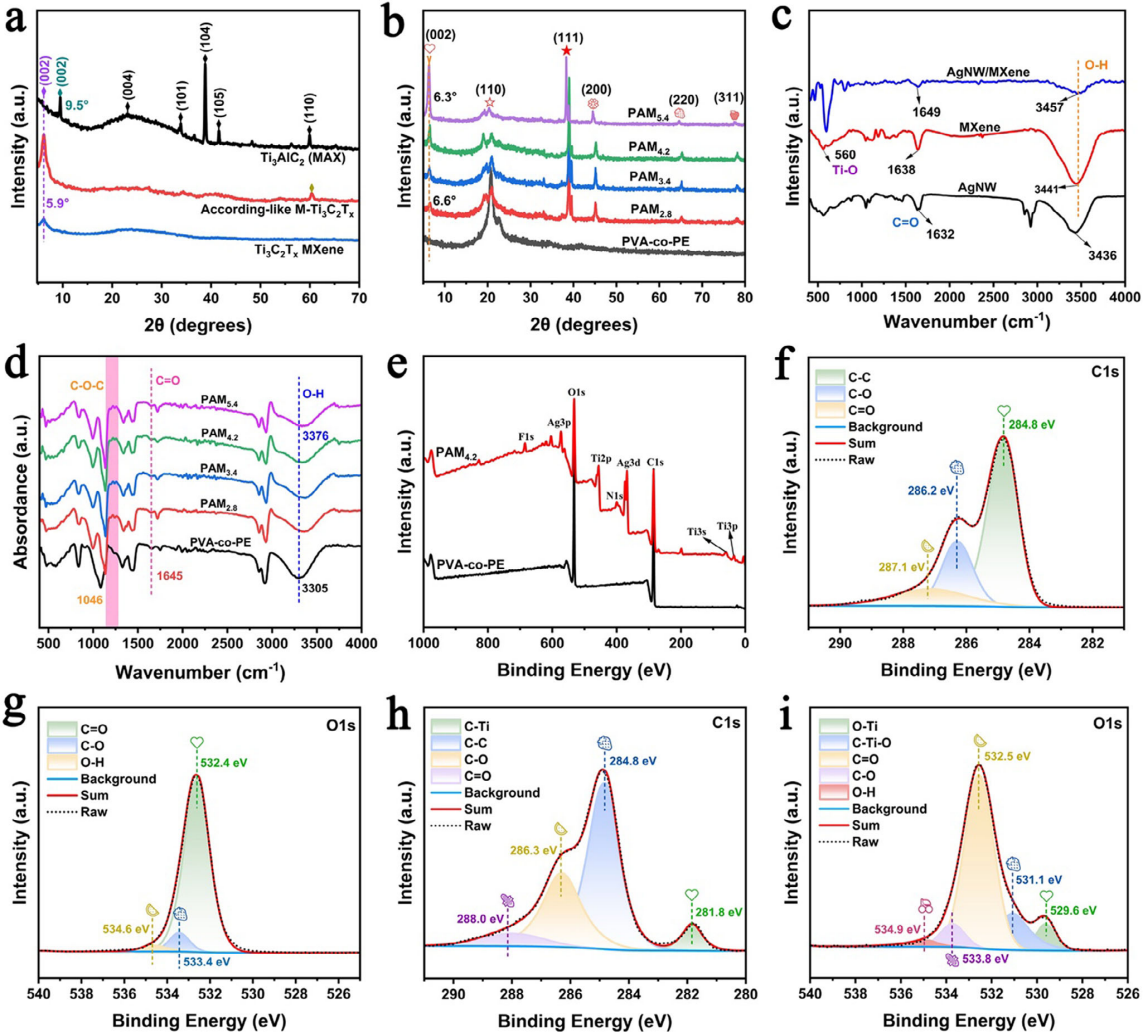
a) XRD patterns of MAX and Ti_3_C_2_T_x_ MXene. b) XRD patterns of different PAM_x_. c) FTIR spectra of AgNW, MXene, and AgNW/MXene. d) FTIR spectra of different PAM_x_. e) XPS wide‐scan spectra of PVA‐co‐PE and PAM_4.2_. High‐resolution XPS spectra of f) C 1s and g) O 1s for the PVA‐co‐PE film. High‐resolution XPS spectra of h) C 1s and i) O 1s for PAM_4.2_.

**Figure** **3**a shows the XRD patterns of Ti_3_AlC_2_ (MAX) for the preparation of Ti_3_C_2_T_x_ MXene. The (104) crystal plane almost completely disappeared, and the peak corresponding to the (002) crystal plane of Ti_3_C_2_T_x_ MXene at 9.5° shifted to 5.9°. This was due to the expansion of the interlayer spacing of MXene caused by the etching of the Al layer, further confirming the successful preparation of MXene.^[^ ^13^, ^53^ ^]^ Figure 3b shows the XRD patterns of the PVA‐co‐PE nanofiber film and different PAM_x_. The (002) crystal plane of all PAM_x_ was at 6.3°, indicating that MXene was successfully crosslinked onto the nanofiber film. The (111), (200), (220), and (311) crystal planes are characteristic of AgNW, and their corresponding peaks confirmed the successful preparation of AgNW and their successful crosslinking with the nanofibers.^[^ ^54^ ^]^ In addition, as the AgNW/MXene content increased, the peak intensity of the (002) crystal plane in PAM_x_ increased. Moreover, the increase in the AgNW/MXene content increased the crystallinity of the composite film and enhanced its mechanical properties. Figure 3c shows the Fourier transform infrared (FTIR) spectra of AgNW, MXene, and AgNW/MXene. MXene exhibited characteristic peaks at 560 and 1638 cm^−1^ corresponding to Ti─O and C ═ O, respectively. The FTIR spectrum of the AgNW showed a stretching vibration peak at 1632 cm^−1^, attributed to the C ═ O group of polyvinylpyrrolidone (PVP) on the AgNW during their preparation. Compared to the peak at 1632 cm^−1^ in the FTIR spectra of AgNW and MXene, the characteristic peak position of C ═ O in the FTIR spectrum of AgNW/MXene shifted to 1649 cm^−1^ owing to changes in the chemical environment, and the characteristic peak of hydroxyl (O─H) also shifted from 3436 to 3457 cm^−1^. This is because hydrogen bonds were formed between the C ═ O groups on the AgNW and the abundant oxygen‐containing functional groups (O─H) on the surface of MXene. Figure 3d and Figure S7 (Supporting Information) show the FTIR spectra of the PVA‐co‐PE film and different PAM_x_. PAM_x_ exhibited a stretching vibration peak at 1046 cm^−1^, corresponding to the ether bond (C─O─C) formed by the chemical crosslinking of PVA‐co‐PE nanofibers with glutaraldehyde. The characteristic peaks at 1645 cm^−1^ and 3376 cm^−1^ correspond to the vibration peak of the carbonyl group (─C ═ O) and the stretching vibration of ─OH, respectively. As the content of AgNW/MXene increased, the position of the O─H group characteristic peak in PAM_x_ shifted to 3376 cm^−1^ compared to the 3305 cm^−1^ peak in PVA‐co‐PE, indicating that hydrogen bonds were formed between PVA‐co‐PE, AgNW, and MXene, which led to the formation of many new O─H groups.

Figure 3e–i present the XPS wide‐scan spectra and high‐resolution C 1s and O 1s spectra of the PVA‐co‐PE film and different PAM_4.2_. The addition of AgNW/MXene introduced various polar groups, including oxides(─O─), fluorine(─F), and hydroxyl groups(–OH), into the composite film. Figure 3e shows the characteristic peaks corresponding to the Ti2p and Ti3p orbitals of MXene and the Ag3d and Ag3p orbitals of the AgNW. The high‐resolution C 1s spectrum of PAM_x_ (Figure 3h) exhibits peaks at 281.8, 284.8, 286.3, and 288.0 eV, assigned to the C─Ti, C─C, C─O, and C ═ O groups, respectively. The presence of C─Ti and C ═ O indicated the abundance of polar groups in PAM_x_, where C─Ti originated from MXene, confirming that MXene remained intact during preparation. Compared with those in the high‐resolution C 1s spectrum of the PVA‐co‐PE film (Figure 3f), the binding energies of C─O and C ═ O in PAM_x_ were shifted from 286.2 eV and 287.1 eV to 286.3 eV and 288.0 eV, respectively. This shift reflects the changes in the chemical environment of these groups, which are attributed to the chemical crosslinking between PVA‐co‐PE and glutaraldehyde, as well as the formation of abundant hydrogen bonds between PVA‐co‐PE, AgNW, and MXene. The high‐resolution O 1s spectrum of PAM_x_ (Figure 3i) displays peaks at 529.6, 531.1, 532.5, and 534.9 eV, corresponding to the O─Ti, C─Ti─O, C ═ O, C─O, and O─H groups, further confirming the presence of numerous oxygen‐containing polar groups. Compared with the 534.6 eV peak in the high‐resolution O 1s spectrum of the PVA‐co‐PE film (Figure 3g), the binding energy of O–H in PAM_x_ shifted to 534.9 eV, indicating the formation of new O─H groups within the composite film. This result is consistent with the FTIR results. Extensive hydrogen bonding and crosslinking enhanced the interfacial forces between AgNW/MXene and PVA‐co‐PE nanofibers, thereby improving the mechanical properties of the composite film and facilitating the formation of a robust conductive network.

### 2.4 Electromagnetic Shielding Performance

**Figure 4:**
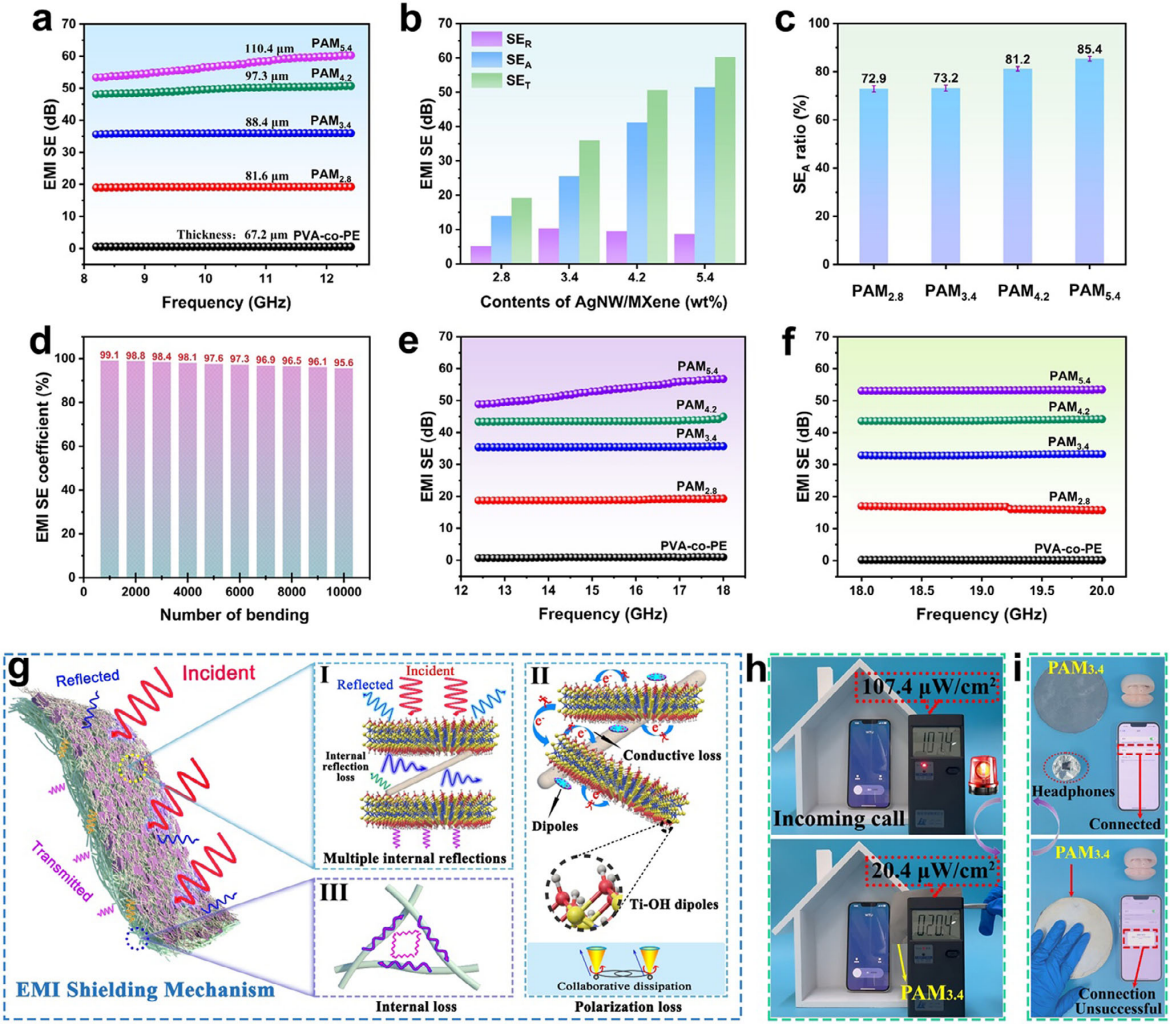
a) EMI SE of different films. b) EMI SE of PAM_x_ with different contents of AgNW/MXene. c) EMW absorption rate of different PAM_x_. d) EMI efficiency of PAM_5.4_ at different numbers of bends. e) EMI SE of the different PAM_x_ films in Ku band. f) EMI SE of the different PAM_x_ films in K band. g) EMI mechanism of PAM_x_. h) EMI SE of PAM_3.4_ on the safe house. i) PAM_3.4_ shielding Bluetooth earphones.

**Figure** **4**a,b shows the EMI SE of different PAM_x_ in the 8.2–12.4 GHz (X‐band) range. The EMI SE increased significantly with increasing AgNW/MXene content. When the AgNW/MXene content was only 5.4 wt.% (PAM_5.4_) and the thickness was 110.4 µm, the composite film exhibited a total EMI SE of up to 60.2 dB in the X‐band, with an absorption loss (SE_A_) as high as 51.4 dB, achieving efficient absorption of ≈85.4% of EM waves (Figure 4c). Further analysis showed that, as the AgNW/MXene content increased, the SE_A_ of PAM_x_ increased significantly, whereas the reflection loss (SE_R_) remained relatively unchanged, as shown in Figure 4b and Figure S8 (Supporting Information). This indicates that the excellent EMI SE of PAM_x_ originates primarily from the enhancement of SE_A_, suggesting that PAM_x_ exhibits low reflection characteristics in its EMI SE. Subsequently, considering the EMI SE of PAM_x_ under multiple bending cycles, the long‐term stability of PAM_5.4_ was investigated. As shown in Figure 4d, PAM_5.4_ retained 95.6% of its EMI SE after 10 000 bending cycles at a strain of 90%. The excellent stability of the composite film is attributed to the enhanced hydrogen bonding between nanofibers, AgNW, and MXene, while the chemical crosslinking between nanofibers further strengthens the mechanical stability of the composite film. Additionally, PAM_x_ demonstrated excellent EMI SE in the wide frequency ranges of 12.4–18.0 GHz (Ku‐band) and 18–20 GHz (K‐band). When the AgNW/MXene content was 5.4 wt.%, the EMI SE of PAM_5.4_ exceeded 50 dB in both frequency bands. The excellent EMI SE results from the synergistic EMI shielding mechanism of the heterostructure and the porous structure of the nanofibers.

In summary, the low‐reflection, heterostructured composite film prepared in this study achieved excellent EMI SE (60.2 dB) across an ultrawide frequency range. Figure 4g illustrates the EM shielding mechanism. When incident EMW reach the surface of the composite film, although a portion of the incident waves is reflected, most of them penetrate into the interior and interact with the AgNW/MXene conductive network layer on the surface of the heterostructure. The induced electric field interacts with high‐density charge carriers (such as free electrons and holes), leading to EMW attenuation through the combined effects of eddy current, polarization, and resonant losses, ultimately achieving strong absorption of EMWs (Figures 4gI,II). Additionally, multiple internal reflections between adjacent MXene nanosheets further promote the dissipation and attenuation of EMW, leading to complete absorption (Figure 4gI). AgNW and MXene contain abundant polar groups. The polar groups in the AgNW originate from PVP and form local defects on the surface and edges, inducing asymmetric charge density distributions. This generates local dipoles (C ─ O, Ti─OH) that undergo directional rotation under an EM field, producing relaxation loss and enhancing the EM shielding performance (Figure 4gII). Furthermore, the special porous structure further increases multiple internal reflection losses of the EMW (Figure 4gIII). The remaining portion of EMW continues to propagate through the film into the nanofiber layer, where its special porous structure further increases the multiple internal reflection losses of EMW (Figure 4gIII). Ultimately, a negligible portion of EMW penetrates the composite film and continues to propagate. These results indicate that the excellent EM shielding performance of the PAM_x_ composite films mainly stems from the reflection‐absorption‐multiple reflection mechanism of EM waves by the heterostructure and the porous structure of the nanofiber composite film.

To directly verify the excellent EM shielding performance of PAM_x_, an EM radiation detector was used to measure the shielding effect of PAM_3.4_ on EM radiation from electronic devices, as shown in Figure 4h and Movie S1 (Supporting Information). In an unprotected room, the radiation power density of a smartphone during an incoming call is 107.4 µW cm^−2^, which exceeds the safety threshold and triggers the alarm light of the detector. When protected by PAM_3.4_, the radiation power density drops to 20.4 µW cm^−2^, and the alarm light turns off, which is because the composite film absorbed most of the EMW generated during the smartphone's incoming call. Figure 4i and Movie S2 (Supporting Information) illustrate the shielding effect of PAM_3.4_ on the Bluetooth signals. First, a simple container was fabricated using aluminum foil, and a Bluetooth earphone was placed inside it. A small hole was opened at the top to maintain normal connectivity between the smartphone and the earphones. When the hole was sealed with PAM_3.4_, the Bluetooth earphone was disconnected from the smartphone, indicating that PAM_3.4_ had completely blocked the wireless signal transmission, preventing the smartphone from receiving signals, and its shielding mechanism is shown in Figure 4g. These results demonstrate that PAM_x_ exhibits outstanding EMI shielding performance.

**Figure 5:**
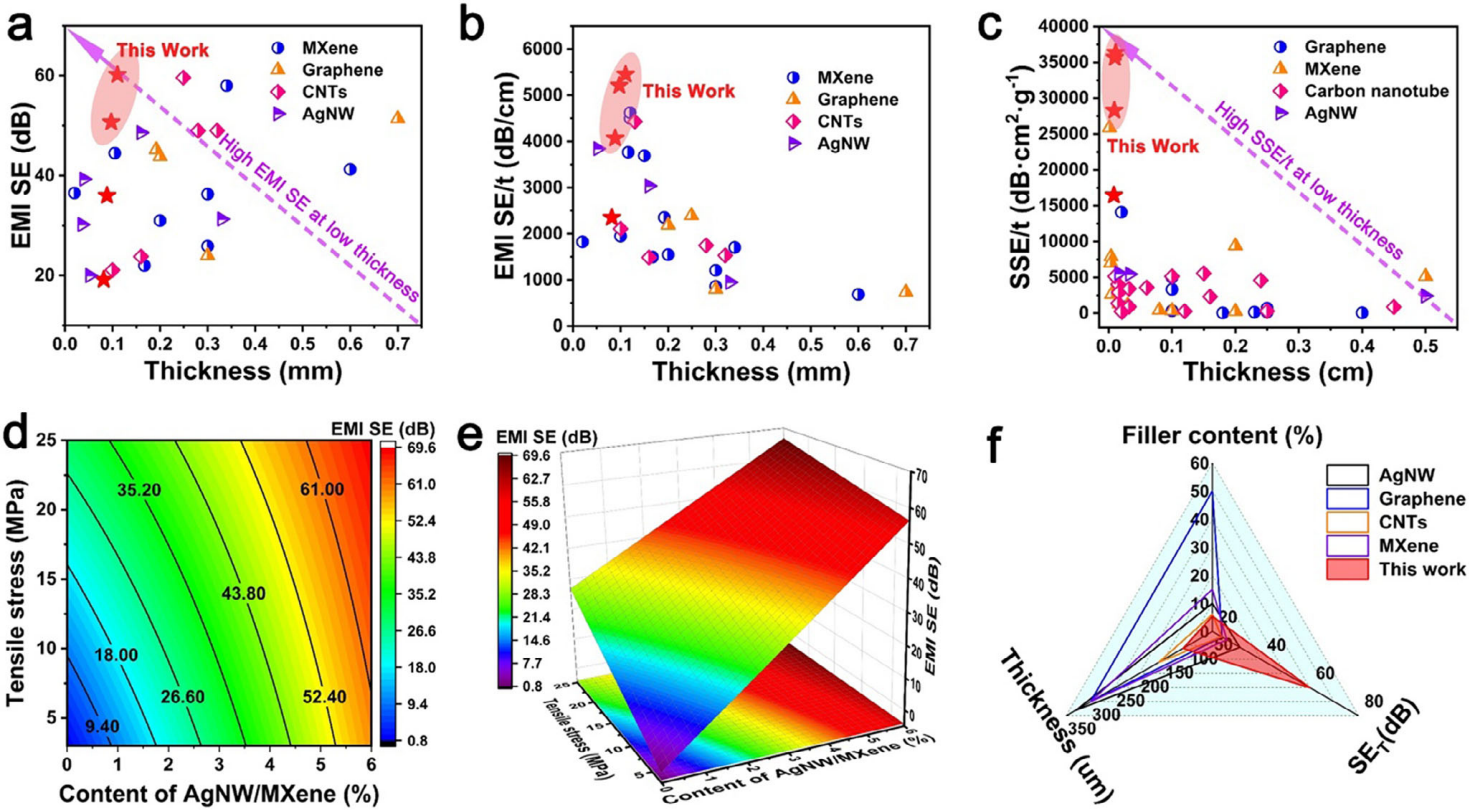
PAM_x_ performance compared with that achieved in previous work. a) EMI SE, b) EMI SE/t, and c) SSE/t. d) Contour map and e) EMI SE surface response and tensile strength as AgNW/MXene content is varied. f) Compared with the reported films,^[55–58]^ the PAM_x_ demonstrated excellent EMI SE.

The thickness and weight of composite films are critical criteria for evaluating high‐performance EMI shielding materials, particularly in applications such as smart electronics, wearable devices, and aerospace engineering.^[^ ^27^, ^31^, ^40^ ^]^ Considering the influence of thickness on the EMI SE, the EMI SE was normalized to EMI SE/t to eliminate the effect of thickness on the material performance evaluation. The normalization of the absolute shielding efficiency (SSE/t) is a key criterion for assessing whether a composite film exhibits efficient lightweight EM shielding performance. As shown in **Figures** **5**a–c and Table S1 (Supporting Information), compared with other reported materials, PAM_x_ ranked among the top in terms of EMI SE, EMI SE/t, and SSE/t in the comparison chart. In this study, when the AgNW/MXene content was as low as 5.4 wt.% and the thickness was only 110.4 µm, PAM_5.4_ exhibited EMI SE, EMI SE/t, and SSE/t values as high as 60.2 dB, 5452.9 dB·cm^−1^, and 36352.7 dB·cm^2^·g^−1^, respectively. This excellent performance primarily benefits from the heterostructured AgNW/MXene layer of PAM_x_, which allows most of the EM waves to enter the interior of the composite film for attenuation. The porous structure inside the composite film further enhances the multiple reflections and absorption losses of EM waves, ultimately achieving complete absorption of EM waves. The PAM_x_ prepared in this study realizes multi‐stage absorption‐reflection loss through a synergistic EMI shielding mechanism between the heterostructured AgNW/MXene layer and the internal porous structure of the nanofiber film, which significantly improves the EMI SE and provides a simple and effective strategy for preparing composite films with low reflection, light weight, and high EMI SE.

The relationship between the tensile stress of the composite film, AgNW/MXene content, and EMI SE (the theoretical equation follows Equations (S11) and (S12), Supporting Information) was investigated, and the results are shown in Figure 5d.e. At low filler loadings (<10 wt.%), the tensile stress of the composite film exhibited an approximately linear increase with increasing AgNW/MXene contents, further confirming the formation of hydrogen bonds in the composite film. In addition, the EMI SE increased with increasing AgNW/MXene contents. Figure 5f shows a radar chart comparing PAM_x_ with previously reported EM shielding materials.^[^ ^55^, ^56^, ^57^, ^58^ ^]^ These results demonstrate that PAM_x_ exhibits excellent comprehensive performance. Therefore, reducing their production costs and expanding their application in smart wearable devices and flexible electronics are of great significance.

### 2.5 Joule Heating Property

**Figure 6:**
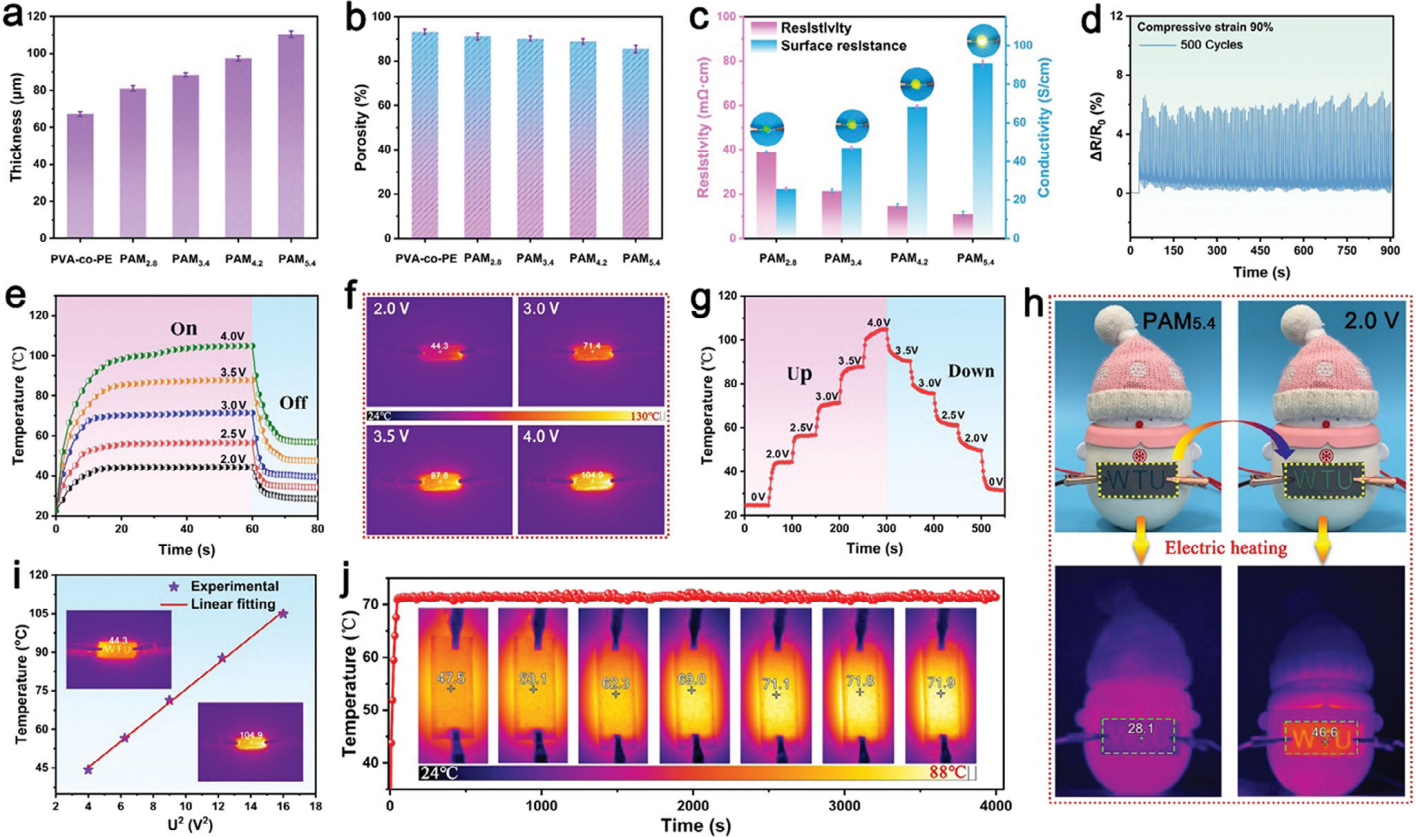
a) Thicknesses and b) porosities of different PAM_x_. c) Electrical conductivities. d) Resistance change rate of PAM_x_ after 500 bending cycles. e) Time‐dependent surface temperatures of the PAM_5.4_ film. f) Surface temperatures of the PAM_5.4_ film at different voltages at 55 s. g) Tailored surface temperatures of the PAM_5.4_ film. h) Digital and IR camera images of the electrical heaters in a wearable thermotherapy device operating at a supplied voltage of 2.0 V. i) Experimental data and linear fitting of the saturation temperature versus U^2^. j) Long‐term time‐temperature curve at a constant voltage of 3.0 V for the PAM_5.4_ film.

In addition to its excellent EM shielding performance, the composite film exhibited outstanding Joule heating behavior, showing great potential for application in wearable thermal textiles and electronic fabrics. As shown in **Figure** **6**a and based on the thickness distribution of the various films, the thickness of the PVA‐co‐PE film was 67.2 µm. With increasing AgNW/MXene content, the thickness of PAM_x_ increased gradually. This was attributed to the increase in density caused by the introduction of AgNW/MXene and the formation of hydrogen bonds among the active groups of PVA‐co‐PE, AgNW, and MXene. These interactions enhance interfacial adhesion within the film and facilitate the construction of efficient conductive and thermal pathways. Figure 6b shows the porosities of the films. All PAM_x_ films exhibited porosity values exceeding 85% owing to the inherently porous structure of the nanofiber matrix and the relatively low loading of AgNW/MXene. Figure 6c shows the resistivity and conductivity of various PAM_x_ films. As the AgNW/MXene content increased, the conductivity of the composite films improved significantly. At 5.4 wt.% AgNW/MXene, the conductivity of the PAM_5.4_ film reached 90.6 S cm^−1^, compared to zero for the PVA‐co‐PE film. With this conductivity enhancement, the brightness of the connected LED bulb increased (Figure 6c). The electrical stability of the PAM_x_ films under mechanical deformation was investigated. As shown in Figure 6d and Movie S3 (Supporting Information), under 90% compressive strain and 500 loading‐unloading cycles, the resistance variation remained below 7%, demonstrating excellent mechanical‐electrical durability. Furthermore, under a low operating voltage of 2.5 V, PAM_x_ successfully powered an LED bulb and a small fan, which continued to operate stably during the bending and twisting of the film, with no noticeable change in brightness or fan speed (Movies S4 and S5, Supporting Information). These results confirm that the AgNW act as conductive bridges, linking the MXene sheets and nanofibers via hydrogen bonding, thereby forming a stable and interconnected conductive network. This structure imparts the composite film with exceptional electrical conductivity and mechanical stability, thereby enabling its application in flexible and wearable electronic systems. Moreover, the distribution of conductive media in the limited space on the surface greatly improves the utilization rate of conductive media and helps to solve the bottleneck of low filling and high conductivity.

The Joule heating performance of PAM_x_ was evaluated by varying the voltage and measuring the surface temperature. Figure 6e shows the time‐temperature curves of PAM_5.4_ under voltages of 2.0–4.0 V. At 2.0 V, the surface temperature of PAM_5.4_ rapidly rose to 44.1°C within 20 s, reaching a comfortable temperature for the human body, making it suitable for warm fabrics and thermotherapy applications. As the voltage increased, the resistivity of the composite film remained nearly constant while the current increased, generating more Joule heat and significantly raising the steady‐state saturation temperature, as shown in Figure 6e,f. Figure 6g shows the online regulation of the surface temperature of the PAM_x_ film within a voltage range of 0–4.0 V, indicating its excellent fast Joule heating response. Figure 6h illustrates the visual Joule heating effect of PAM_5.4_. At 2.0 V, as the surface temperature rises, the “WTU” pattern formed by thermal‐sensitive materials adhered to the PAM_5.4_ film changes from dark blue to green (Movies S6, Supporting Information). Additionally, compared with the control group, at 2.0 V, the infrared thermal image clearly shows a significant temperature increase around the PAM_5.4_ film on the “snowman” toy, as indicated by a distinct WTU pattern (Figure 6h), further demonstrating the broad application potential of PAM_x_ in smart wearable textiles and Joule heaters. Figure 6i shows the linear fitting results between the surface temperature of PAM_x_ and the square of the voltage (U^2^). These results indicate that the surface temperature is nearly linearly related to U^2^, which is consistent with the literature.^[^ ^13^, ^59^ ^]^ Figure 6j shows the long‐term stability test results of the Joule heating of PAM_5.4_. At 3.0 V, the saturation temperature of the composite film surface was ≈70 °C, with stable operation exceeding 66 min. In summary, the heterostructured PAM_x_ prepared in this study exhibits excellent Joule‐heating performance, along with excellent reliability and stability for practical applications.

### 2.6 Mechanical Property

**Figure 7:**
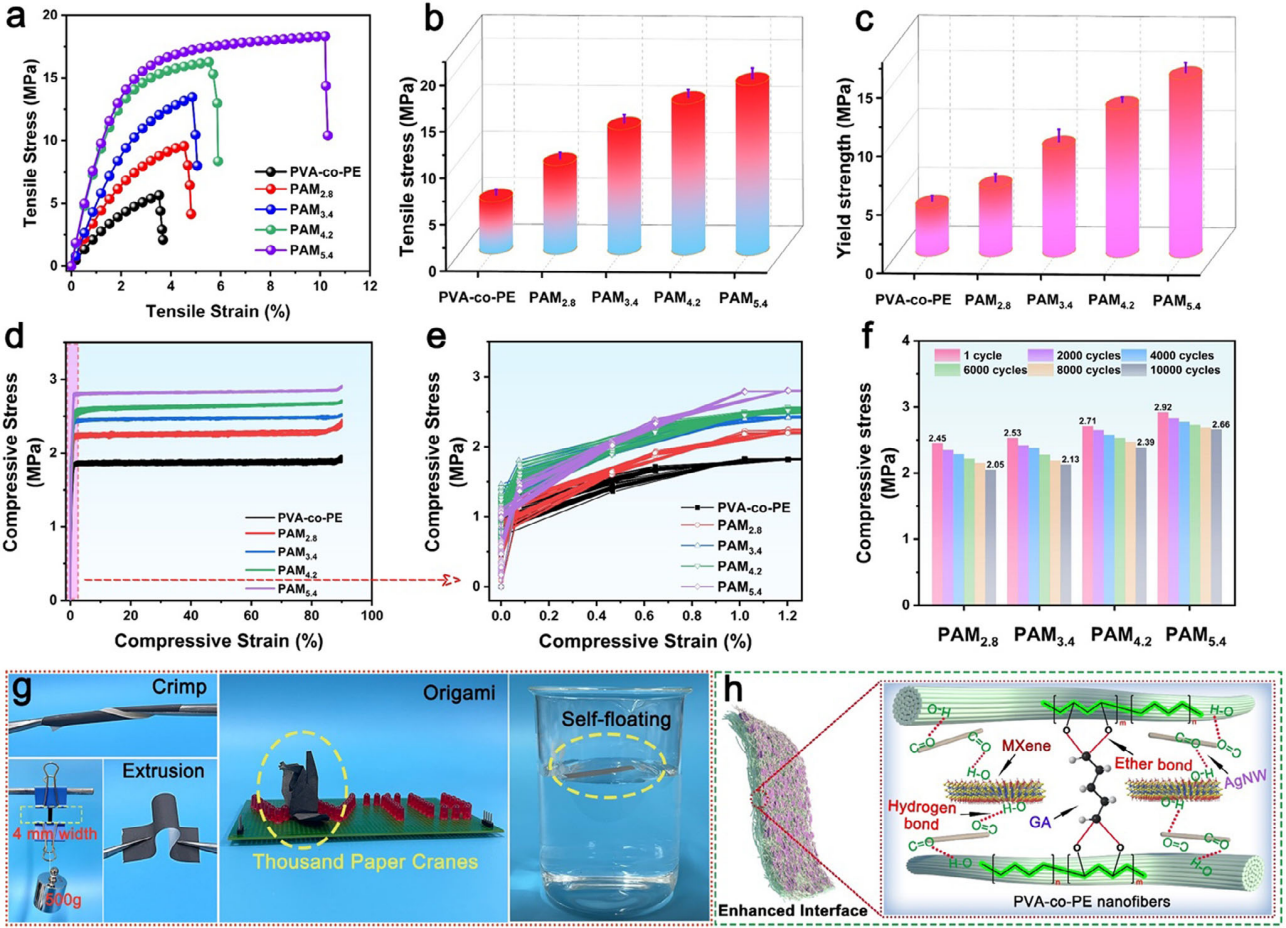
a–c) Tensile strength of PAM_x_ nanofiber films. d,e) Low‐ and high‐magnification cyclic compression curves of PAM_x_ for 100 cycles. f) Stress values at different numbers of compression cycles. g) Images of the twisting, stretching, extrusion, folding, and self‐floating of the PAM_4.2_ film. h) Mechanism diagram for enhancing the mechanical properties of PAM_x_.

The PAM_x_ film prepared in this study shows potential for applications in flexible sensing and smart wearables, necessitating further evaluation of its mechanical properties. **Figure** **7**a–c shows the tensile performance of different films. As the AgNW/MXene content increased, both the tensile stress and strain of PAM_x_ improved significantly. When the AgNW/MXene content was as low as 5.4 wt.%, the tensile stress of PAM_5.4_ reached 18.33 MPa (Figure 7b), representing a 224% improvement in tensile performance compared to that of the control sample. Additionally, as shown in Figure 7c, the yield strength of the PAM_x_ film also increased significantly, indicating enhanced internal interfacial forces and improved toughness. Figure 7d,e presents the compressive stress‐strain curves of the different films after 100 cycles of compression at a strain of 90%. With increasing AgNW/MXene contents, the compressive stress of the composite films increased, and the compression curves after multiple cycles nearly overlapped, demonstrating the excellent cyclic compression stability of PAM_x_. Figure 7f shows the compressive stress of PAM_x_ under different numbers of compression cycles. After 10 000 compression cycles, all PAM_x_ films maintained a compressive stress retention rate of over 83.6%, with PAM_5.4_ achieving an outstanding retention rate of 91.1%, highlighting its superior cyclic compression stability. Figure 7g displays optical images of PAM_4.2_, showing its high flexibility, high tensile strength, and lightweight characteristics.

In summary, PAM_x_ exhibits excellent mechanical properties that are primarily attributed to the interactions between its components. First, the abundant ─OH groups on the surface of the PVA‐co‐PE nanofibers react chemically with the ─C ═ O groups of the crosslinking agent (glutaraldehyde) to form ether bonds (C─O─C). Additionally, the PVP on the surface of AgNW contains numerous active groups (C ═ O), whereas the ─OH groups on the surfaces of MXene and PVA‐co‐PE nanofibers interact chemically to form O–H bonds. The AgNW acts as an intermediate medium for constructing a robust 3D network structure between MXene and PVA‐co‐PE nanofibers, further enhancing the mechanical properties of PAM_x_, as illustrated in the reinforcement mechanism shown in Figure 7h.

### 2.7 Sensing Property

**Figure 8:**
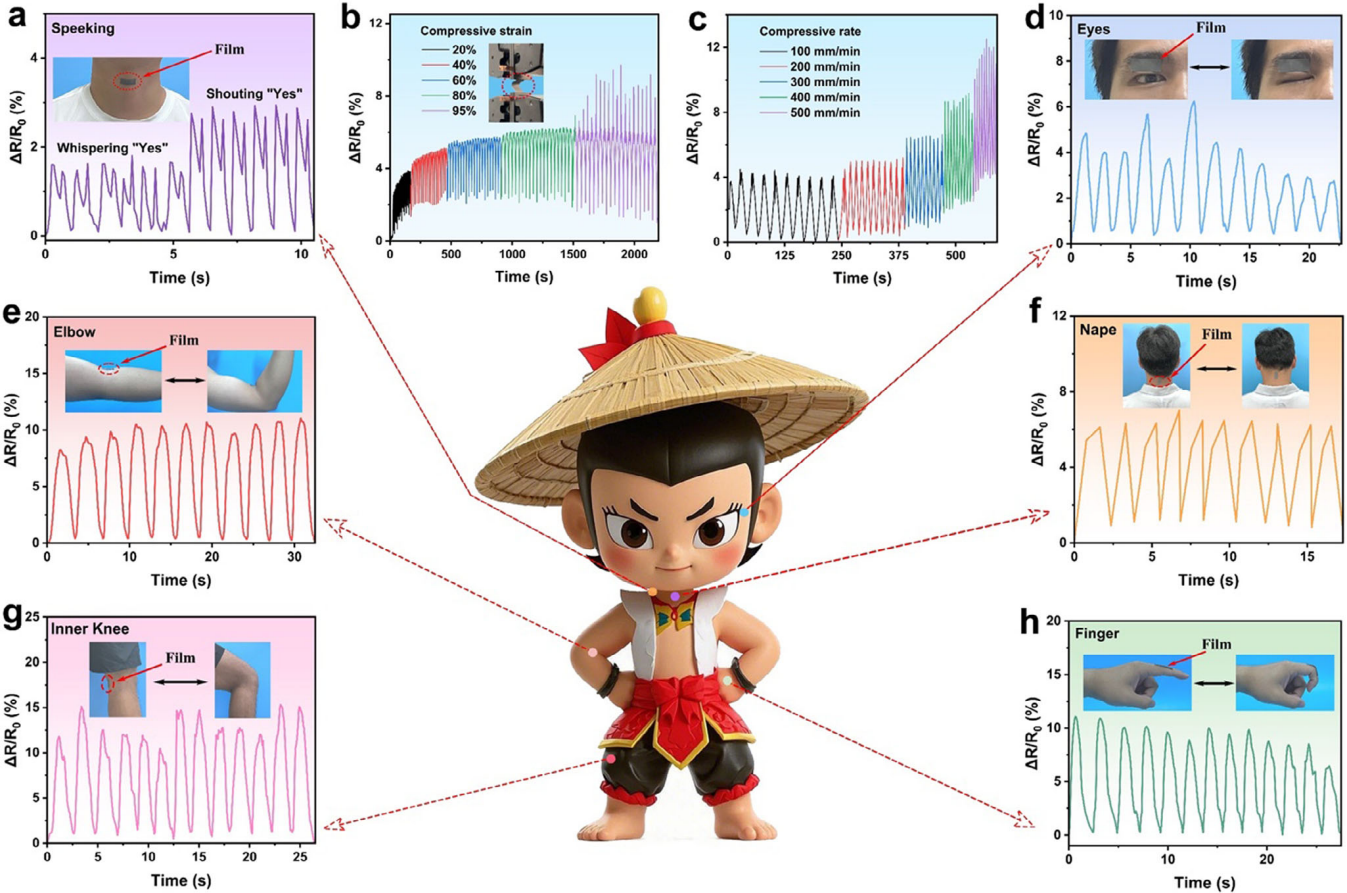
a) Rate of resistance change of the PAM_x_ sensor upon the vocalization of “yes.” b) Resistance change rate at different compressive strains. c) Resistance change rate at 80% strain under different tensile and compressive rates. d) Sensing curve of the PAM_x_ sensor during eye blinking, and schematic of e) elbow, f) nape, g) knee, and h) finger joint motion sensing. (The cartoon character was generated by Jimeng AI).

The excellent electrical conductivity and mechanical properties of the heterostructured composite films prepared in this study endow them with the potential for application in flexible sensing and human motion monitoring. The relative resistance changes under different compressive deformations and rates were investigated to evaluate the sensing characteristics of the composite film as a strain sensor, as shown in **Figure** **8**b,c, respectively. As the strain and compression rate increased, the relative resistance changes of PAM_x_ increased but remained below 13%. This is likely because the compressive strain increased the contact area between the AgNW, MXene, and PVA‐co‐PE nanofibers, thereby improving electron conduction. Additionally, PAM_x_ exhibits distinguishable resistance changes of varying magnitudes under different strains and compression rates, producing distinct signals.^[^ ^11^ ^]^ To further explore the sensing capability of the composite film sensor, real‐time electrical signals from the PAM_x_ sensor attached to the human body were collected using a digital source meter. As shown in Figure 8a, when the composite film sensing system was applied to the throat, the sensor effectively converted vibrations from speech into the corresponding electrical signals, demonstrating the high sensitivity of the PAM_x_ sensor to human motion. Subsequently, when the composite film sensor was attached to the human eye (Figure 8d), elbow (Figure 8e), neck (Figure 8f), inner knee (Figure 8g), and finger (Figure 8h), the relative resistance changes exhibited a stepwise increasing or decreasing pattern with the normal movements of each body part. This indicates that the sensor can accurately respond to the bending angles and frequencies of different joints with clearly distinguishable response signal amplitudes. In summary, the PAM_x_ films prepared in this study exhibited an excellent sensing performance, highlighting their broad application prospects in flexible sensing, medical monitoring, and other related fields.

### 2.8 Photothermal Properties

**Figure 9:**
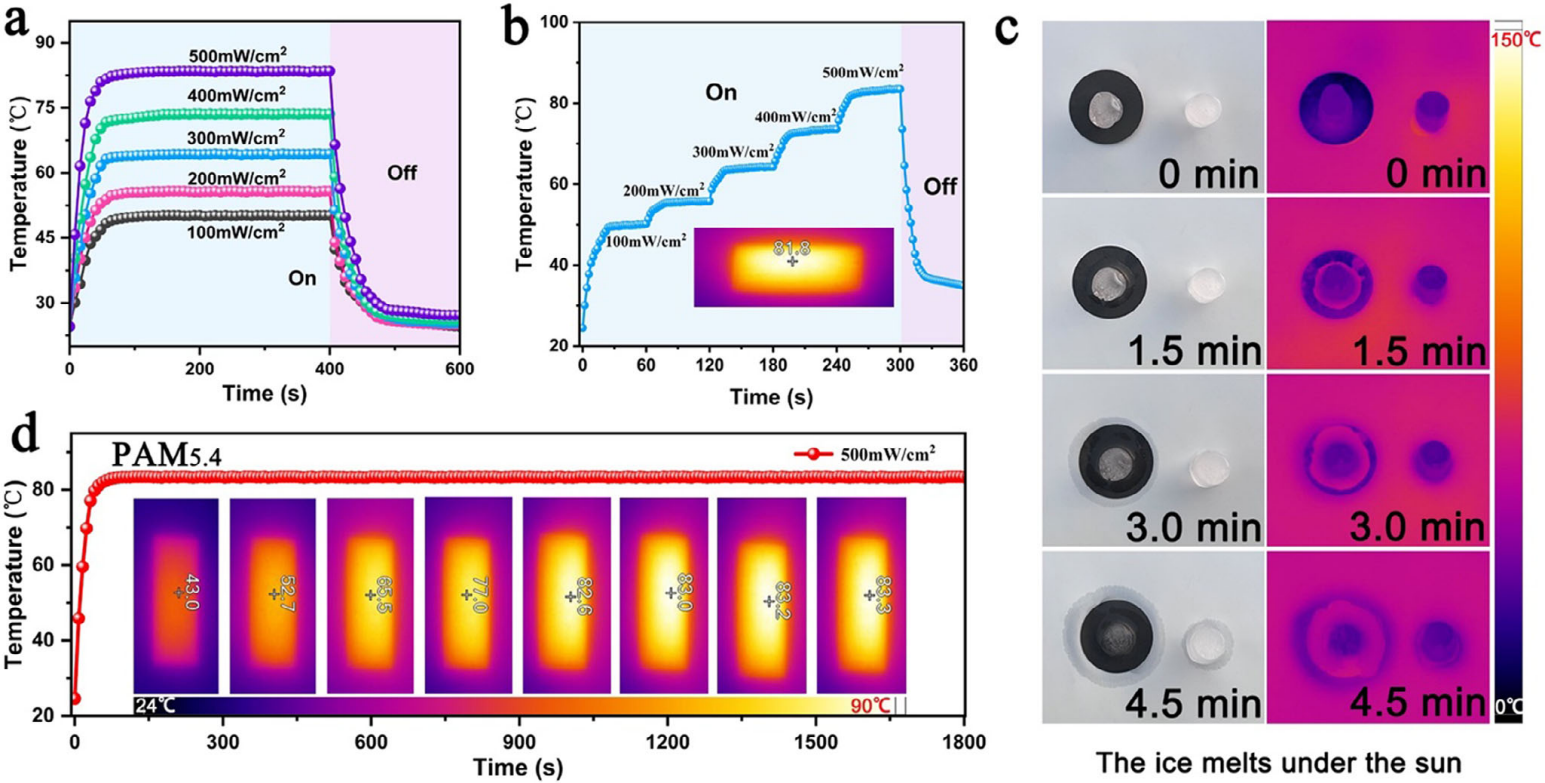
Photothermal conversion performance of PAM_5.4_. a) Temperature profiles of PAM_5.4_ over time under different light power densities. b) Tailored surface temperatures of PAM_5.4_. c) Images of the ice‐melting process under sunlight using PAM_5.4_. d) Surface temperature variation at a light power density of 500 mW cm^−2^.

Extensive studies have shown that both AgNW and MXene exhibit excellent photothermal conversion capabilities.^[^ ^60^, ^61^ ^]^ Therefore, the introduction of AgNW/MXene endows the composite film with superior photothermal conversion performance, and the porous structure of PAM_x_ further enhances the absorption of incident light. **Figure** **9**a shows the variation in the surface temperature of PAM_5.4_ over time under different light power densities. As the light power density increased, the surface saturation temperature of the composite film increased significantly, exhibiting a rapid response to changes in the light power density gradient. At a light power density of 300 mW cm^−2^, PAM_5.4_ rapidly reached a steady‐state temperature (≈64.1°C) within 60 s, and the temperature quickly dropped to room temperature after the light source was turned off. In addition, the surface temperature of the composite film was dynamically regulated by adjusting the power density (Figure 9b). Figure 9d illustrates the long‐term stability of the composite film surface. The results showed that at a light power density of 500 mW cm^−2^, PAM_5.4_ maintained a thermal equilibrium temperature for at least 1800 s, demonstrating excellent photothermal stability and reliability. Figure 9c presents the results of a photothermal ice‐deicing performance test for PAM_5.4_ under sunlight (ambient temperature of 37°C). The results indicate that with increased illumination time, the ice on the composite film melted almost completely after only 4.5 min, whereas the ice in the control group had just begun to thaw. In summary, the heterostructured PAM_x_ prepared in this study exhibited outstanding photothermal conversion performance, holding potential for application in ice de‐icing and thermal insulation in cold regions.

### 2.9 Hydrophobic and Flame‐Retardant Properties

**Figure 10:**
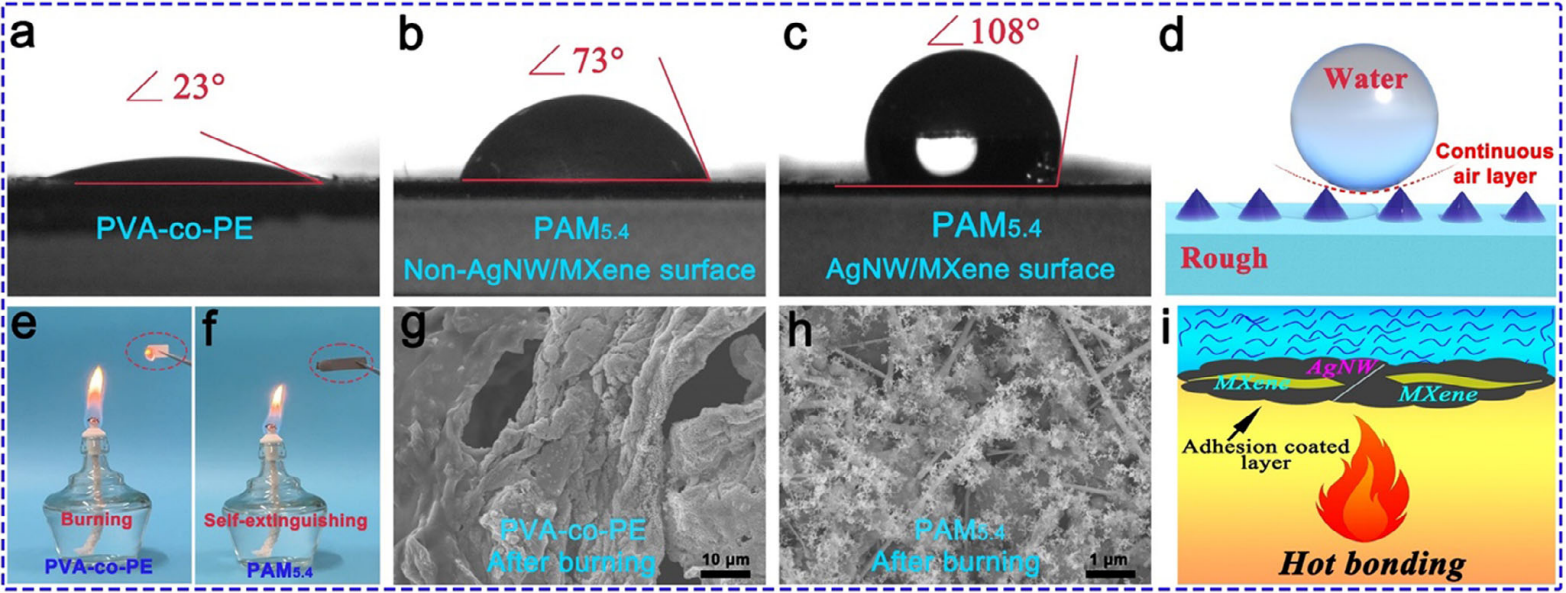
a–c) Water contact angles of PVA‐co‐PE and PAM_5.4_ films. d) Schematic of the enhanced hydrophobicity mechanism of PAM_x_. e,f) Optical images of flame tests of different films. g,h) SEM images of the films after combustion. i) Self‐extinguishing combustion mechanism of PAM_x_.

With the rapid development of smart devices and new types of weaponry, high‐performance EMW absorbing materials are often required to enable their applicability in extreme environments.^[^ ^54^ ^]^ **Figure** **10**a–c shows the water contact angles of PVA‐co‐PE and PAM_5.4_, respectively. The water contact angle of PVA‐co‐PE was 23° (Figure 10a, Movie S7, Supporting Information), indicating hydrophilicity, while the water contact angles on the surfaces of the nanofibers and AgNW/MXene in PAM_5.4_ were 73° and 108°, respectively (Figure 10b,c, Movies S8 and S9, Supporting Information). The excellent hydrophobicity results from the introduction of AgNW/MXene, which increases the surface roughness of the composite film and forms a continuous layer of air between the water droplets and the composite film's surface, as shown in Figure 10d.

Figure 10e,f show the combustion images of the PVA‐co‐PE film and PAM_5.4_, respectively. The PVA‐co‐PE film burned completely (Movie S10, Supporting Information), whereas after ignition, the PVA‐co‐PE nanofibers in PAM_5.4_ adhered rapidly to the AgNW/MXene on the heterostructured surface to form a dense incomplete combustion char layer, delaying the combustion process and achieving a self‐extinguishing effect (Movie S11, Supporting Information). Figure 10g,h show SEM images of PVA‐co‐PE and PAM_5.4_ after combustion. The SEM images of PAM_x_ show that the incompletely burned nanofibers were significantly entangled with AgNW/MXene, forming a flame‐retardant layer and further promoting rapid heat dissipation. This phenomenon was not observed for the PVA‐co‐PE film. The self‐extinguishing mechanism is illustrated in Figure 10i. In summary, the heterostructured PAM_x_ prepared in this study exhibits excellent hydrophobicity and nonflammability, making it an ideal material for achieving efficient EMI shielding in extreme environments.

## 3 Conclusion

In this study, a heterostructured PAM_x_ with low reflectivity was successfully prepared. Synergistic optimization of low AgNW/MXene filler loading and high‐performance EMI shielding was achieved using a two‐step spray‐coating method and a solvent evaporation process, combined with chemical crosslinking and hydrogen bonding. Chemical crosslinking and hydrogen bonding enhance interfacial interactions, thereby constructing a stable 3D conductive network and improving the mechanical properties of PAM_x_. This enables the composite film to maintain its excellent conductivity and stability under repeated bending, stretching, and harsh environmental conditions. The heterostructure facilitates 85.4% of the incident EM waves to enter the film, where high ohmic and resonant losses from the AgNW/MXene conductive network, multiple reflection losses between MXene nanosheets, and scattering effects from the porous structure of the nanofibers significantly reduce the EMW transmission energy. When the AgNW/MXene content was only 5.4 wt.% and the thickness was 110.4 µm, the heterostructured composite film exhibited an EMI SE of 60.2 dB, with an SE_A_ of 51.4 dB, achieving 85.4% EM wave absorption. The EMI SE/t and SSE/t were 5452.9 dB·cm^−1^ and 36352.7 dB·cm^2^·g^−1^, respectively. Notably, through interfacial reinforcement via chemical crosslinking and hydrogen bonding, the tensile strength of PAM_x_ increased to 18.33 MPa, and its EMI SE retention rate remained at 95.6% after 10 000 bending cycles at 90% strain. In addition, PAM_x_ not only demonstrated excellent electrothermal and photothermal conversion performances, such as high Joule heating at low voltages, rapid temperature rise response, fast heat dissipation capability, and stability, but also exhibited excellent flexible sensing performance for human motion monitoring. The composite films exhibited outstanding hydrophobicity and flame retardancy in complex environments. This study provides a simple and efficient preparation method of composite films with low dielectric filler loading and efficient low‐reflection EMI shielding performance by constructing heterostructures while simultaneously exhibiting excellent flexibility, superior thermal management, excellent sensing performance, hydrophobic and flame‐retardant properties, and EMI SE stability. These features endow the composite film with significant application potential in fields such as smart wearable EM protective clothing and flexible sensing devices.

## 4 Experimental Section

Details of the materials, synthesis, and characterization methods are provided in the Supporting Information.

## Conflict of Interest

The authors declare no conflict of interest.

## Supporting information



Supporting Information

Supplemental Movie 1

Supplemental Movie 2

Supplemental Movie 3

Supplemental Movie 4

Supplemental Movie 5

Supplemental Movie 6

Supplemental Movie 7

Supplemental Movie 8

Supplemental Movie 9

Supplemental Movie 10

Supplemental Movie 11

## Data Availability

The data that support the findings of this study are available from the corresponding author upon reasonable request.
