# Supplementary material for: High Electrical Conductivity Induced by Surface Confinement Effect in Heterostructured Multifunctional Nanofiber Composite Films for Low‐Reflection Electromagnetic Interference Shielding
Source: Adv Sci (Weinh). 2025 Jul 29;12(40):e10386. doi: 10.1002/advs.202510386 (PMC12561376; doi:10.1002/advs.202510386)
Supplement: Supplementary file 1 — Supporting Information [file ADVS-12-e10386-s006.docx]

Supporting Information

High Electrical Conductivity Induced by Surface Confinement Effect in Heterostructured Multifunctional Nanofiber Composite Films for Low-Reflection Electromagnetic Interference Shielding

Dechang Tao, Xin Wen, Shuai Ma, Can Chen, Jianing Guo, Wenwen Wang, Kun Yan, Chenguang Yang^*^, and Dong Wang^*^

**1.1. Chemicals and Materials**

A nanofiber suspension was prepared using polyvinyl alcohol-copolymer-polyethylene (PVA-co-PE) nanofibers with a diameter of 200-300 nm. Figure S1 shows the preparation schematic diagram of PAV-co-PE nanofibers, the optical image of the suspension, and the microscopic morphology of the nanofibers. The high atomization pump spray gun (W-71) was purchased from Anest Iwata Corporation of Japan. The Ti_3_AlC_2_ (MAX) powder (99.5%) was purchased from Shanghai Adamas Reagent Co., Ltd. Lithium fluoride (LiF), hydrochloric acid (HCl, 33wt%), potassium hydroxide (KOH), silver nitrate (AgNO_3_), ethylene glycol (EG), copper chloride dihydrate (CuCl_2_·2H_2_O), ferric chloride (FeCl_3_), isopropyl alcohol (IPA), sodium bromide (NaBr) and polyvinylpyrrolidone (PVP, Mw=1300000 g/mol) were purchased from Sinopharm Chemical Reagent Co., Ltd. Polypropylene (PP) nonwoven fabric was purchased from Suzhou Jurui textile strength Co., Ltd. The thermochromic material was purchased from Ruixue New Materials Co., Ltd.

**1.2. Synthesis of Silver Nanowires.**

By magnetic stirring, 1.6 g amount of PVP was fully dissolved in 100 mL EG in a three-necked flask at 150°C for 4 hours. Subsequently, 4 mL of 20 mM CuCl_2_·2H_2_O and 4 mM ferric chloride were added to PVP solution to achieve ultra-high aspect ratio AgNW. A mixture of 40 mL of 0.70 M AgNO_3_ and 4 mM sodium bromide was then added to the solution and left for 2 hours at 150°C. After cooling to room temperature, filter acetone and ethanol with inhalation to wash completely. The obtained AgNW were re-dispersed in deionized water at a concentration of 2.5 mg·mL^−1^ for future use. Optical images of the silver nanowire solution and the microstructure of AgNW are shown in Figure S2.

**1.3. Synthesis of Ti_3_C_2_T_x_ MXene.**

As illustrated in Figure S3, the synthesis procedure of Ti_3_C_2_T_x_ MXene is depicted. Initially, 4g LiF were dissolved in a mixture consisting of 60 ml of HCl and 20 ml of deionized water to prepare the etching solution. With continuous magnetic stirring, 2g of Ti_3_AlC_2_ were introduced into the pre - prepared etching solution and the mixture was stirred at 35℃ for 48 h. Subsequently, the mixture was centrifuged at a speed of 4000 r/min for 5 min, and the resulting product was thoroughly washed with deionized water multiple times until the pH value reached 6. Finally, the supernatant was collected by ultrasound for 30 min and centrifugation at a speed of 3500 r/min for 60 minutes, and a dark green colloidal solution of Ti_3_C_2_T_x_ MXene was obtained.

**1.4. Preparation of nanofiber composites film with heterogeneous structure**

Poly(vinyl alcohol-co-ethylene) (PVA-co-PE) and cellulose acetate butyrate (CAB) are uniformly mixed in a mass ratio of 2:8, and then extruded using a twin-screw extruder with a screw diameter of 28 mm and a length-to-diameter ratio of 35 to prepare new-born island fibers. The precursor fibers are extruded using a spinneret with a diameter of 1.8 mm and 12 holes, and spun under the conditions of 200~220°C, 1.5~2.5 MPa, and a winding speed of 1.5 m/s. After removing the sea phase with acetone, nanofibers are obtained, and the process diagram for the preparation of nanofibers is shown in Fig. S1. Subsequently, PVA-co-PE nanofibers were dispersed in a mixed solution of water and IPA in a certain proportion. Glutaraldehyde (2% volume fraction) and hydrochloric acid were added separately to 100 mL of the prepared dispersion with a nanofiber mass fraction of 2.5 wt%. After thorough stirring, the mixture was spray-coated onto polypropylene (PP) non-woven fabric as the base. After the solvent evaporated, a P-PVA-co-PE nanofiber membrane was obtained. According to the required content of AgNW/MXeneh, a uniformly mixed aqueous solution of AgNW and MXene is obtained through ultrasonic treatment and stirring. Then, the AgNW/MXene is spray-applied onto the P-PVA-co-PE nanofiber membrane via the spraying process. After its solvent evaporates, it is dried and formed to obtain a heterostructured P-PVA-co-PE/AgNW/MXene (P-PAM_x_) nanofiber composite membrane, which is then peeled off from the PP matrix to obtain the heterostructured PAM_x_ composite membrane. Finally, heterostructured PAM_x_ (x represents the content of AgNW/MXene, x = 2.8, 3.4, 4.2, 5.4) composite membranes with AgNW/MXene contents of 2.8 wt%, 3.4 wt%, 4.2 wt%, and 5.4 wt% respectively are prepared, in which the mass ratio of AgNW to MXene is 2:1. A PVA-co-PE nanofiber membrane is prepared using the same process as a comparative sample.The fabrication process of the composite membranes is illustrated in Figure 1a.

**1.5. Material Characterizations**

The microstructure and morphology of AgNW, MXene, and PAM_x_ composite films were characterized using a thermal field emission scanning electron microscope (SEM, JSM - 7800F, Japan) and a transmission electron microscope (TEM, 2100P). The crystal structure was analyzed by an X-ray diffractometer (XRD, Empyrean), the chemical structure was examined via a Fourier transform infrared spectrometer (FTIR, Bruker TENSOR - 27), and the elemental composition and valence state changes were characterized using an X-ray photoelectron spectrometer (XPS, AXIS SUPRA). The electrical conductivity and resistivity of the PAM_x_ composite films were tested and characterized with a two-electric-probe four-point probe tester (RTS - 9), and the porosity of the composite films was characterized by a true density analyzer (AccuPyu II 1340). A vector network analyzer (ZNB 20) and Design - expert 12.0 simulation software were employed to optimize the scheme and explore the internal relationship between the experimental process parameters and EMI SE. The surface temperature of the PAM_x_ composite films under different voltage conditions was photographed and recorded using a Fluke infrared thermal imager (Ti400U). Tensile and compression tests were carried out with an INSTRON universal testing machine (INSTRON - 5967). The resistance changes of the PAM_x_ composite films during the movement of different parts of the human body were characterized using a digital source meter (Keithley 2450), and their sensing performance was also evaluated. The photothermal conversion performance of the PAM_x_ composite films was tested and analyzed using a xenon lamp light source system (PLS - SXE 300/300 UV) and a Fluke infrared thermal imager (Ti400U). The water contact angle of the PAM_x_ composite films was tested and analyzed with an optical contact angle measuring instrument (DSA-30S).

**1.6. Electromagnetic shielding effectiveness: theory and measurement**

The EMI SE performance of PAM_x_ composite films in the frequency range of 8.2–20.0 GHz was analyzed using a vector network analyzer (ZNB 20), including measurements of S_11_, S_21_, S_22_, and S_12_ parameters. Based on these values, the transmission (T), reflection (R), and absorption (A) coefficients, as well as the total shielding effectiveness (SE_T_), reflection loss (SE_R_), absorption loss (SE_A_), and multiple internal reflection loss (SE_M_), were calculated using the following equations:

$\text{R=}{\text{|}\text{S}_{\text{11}}\text{|}}^{\text{2}}\text{=}{\text{|}\text{S}_{\text{22}}\text{|}}^{\text{2}}$ (S1)

$\text{T=}{\text{|}\text{S}_{\text{12}}\text{|}}^{\text{2}}\text{=}{\text{|}\text{S}_{\text{21}}\text{|}}^{\text{2}}$ (S2)

$\text{A=1-R-T}$ (S3)

$\text{SE}_{\text{R}}\text{(dB)=10}\lg\left( \frac{\text{1}}{\text{1-R}} \right)\text{=10}\lg\left( \frac{\text{1}}{{\text{1-|}\text{S}_{\text{11}}\text{|}}^{\text{2}}} \right)$ (S4)

$\text{SE}_{\text{A}}\text{(dB)=10}\lg\left( \frac{\text{1-R}}{\text{T}} \right)\text{=10}\lg\left( \frac{{\text{1-|}\text{S}_{\text{11}}\text{|}}^{\text{2}}}{{\text{|}\text{S}_{\text{12}}\text{|}}^{\text{2}}} \right)$ (S5)

$\text{SE}_{\text{M}}\text{(dB)=20}\text{log}_{\text{10}}\left( \text{1-}\text{10}^{\frac{\text{SE}_{\text{A}}}{\text{10}}} \right)$ (S6)

$\text{SE}_{\text{T}}\text{(dB)=}\text{SE}_{\text{R}}\text{+}\text{SE}_{\text{A}}\text{+}\text{SE}_{\text{M}}$ (S7)

Among them, When SE_A_ ≥ 10 dB, SE_M_ can be neglected. EMI SE depends on the dielectric and magnetic properties. At the same time, the EMI SE/t is normalized to eliminate the influence of thickness. In addition, the specific shielding efficiency (SSE) and SSE/t, taking into account density and thickness, are expressed as follows:

$\text{SSE}\text{=}\frac{\text{EMI SE}}{\text{density}}\text{=}\text{dB}\text{∙}\text{c}\text{m}^{\text{3}}\text{∙}\text{g}^{\text{-1}}$ (S8)

$\text{SSE}\text{/}\text{t}\text{=SSE/thickness=}\text{dB}\text{∙}\text{c}\text{m}^{\text{2}}\text{∙}\text{g}^{\text{-1}}$ (S9)

EMI shielding efficiency (%) is obtained by using the following equation:

$\text{Shield efficiency (\%) =100-(}\frac{\text{1}}{\text{10}^{\text{EMI SE}/\text{10}}}\text{)×100}$ (S10)

**1.7. Optimization model design**

A typical square root optimization model (Eq. S11) is used in this work.

$y^{'}=y$ (S11)

The process sequence is executed according to the main effect. The optimal design software Design-Expert 12.0 was used to obtain the final equation (Eq. S12) according to the actual factors.

$$\text{EMI SE (dB) =-3.04979+10.1531*}\text{AgNW}\text{/}\text{MXene}\text{ content (\%) +1.309384*tensile stress}$$

$\text{(MPa) -0.140445*}\text{AgNW}\text{/}\text{MXene}\text{ content (\%)*tensile stress (MPa)}$ (S12)

**2. Results and discussion**


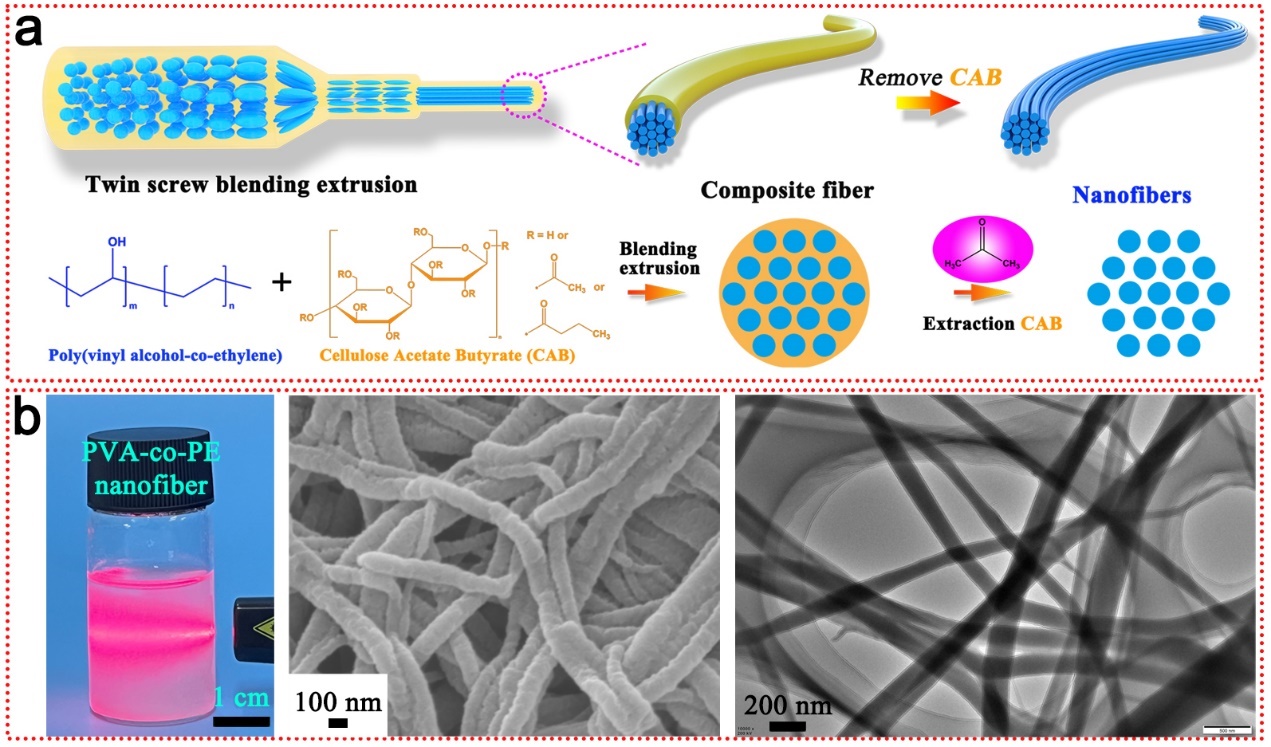


**Figure S1.** (a)Schematic diagram of preparation of PVA-co-PE nanofibers[1]. (b) Optical images of the nanofiber solution and the microstructure of PVA-co-PE nanofibers.


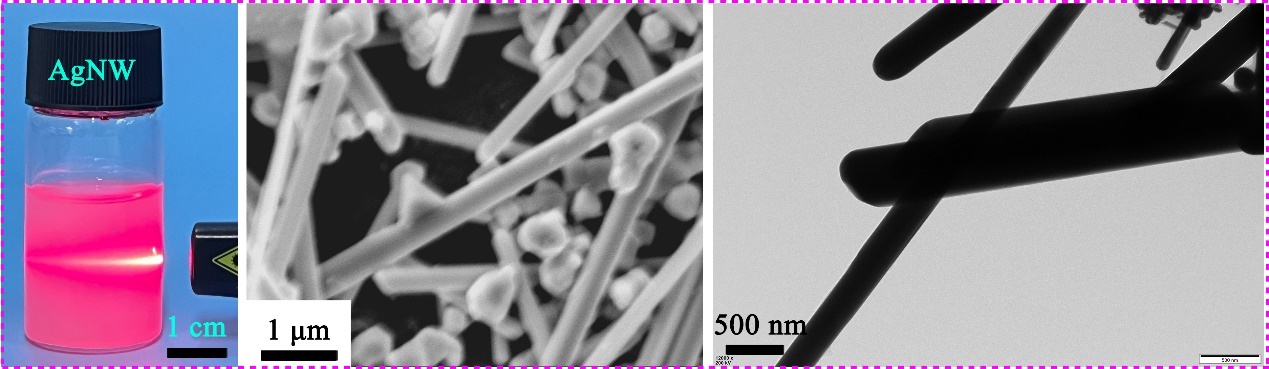


**Figure S2** Optical images of the silver nanowire solution and the microstructure of AgNW.


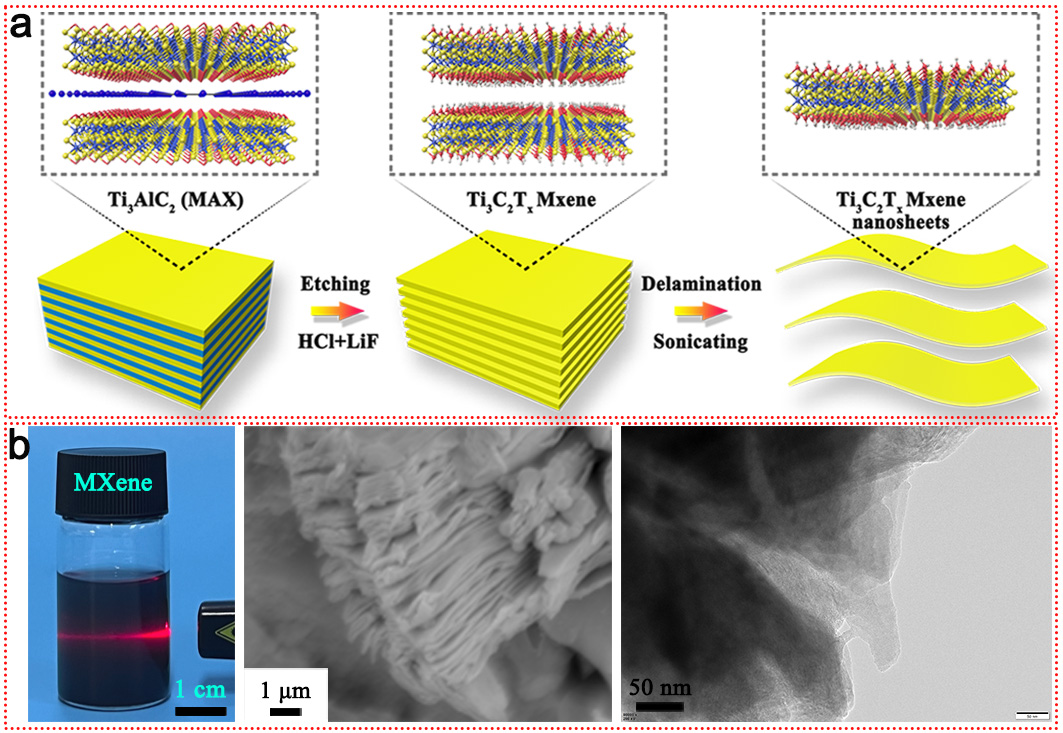


**Figure S3.** (a) Synthesis of Ti_3_C_2_T_x_ MXene *via* the etching and delamination method. (b) Optical images of Ti_3_C_2_T_x_ MXene solution and the microstructure of the Ti_3_C_2_T_x_ MXene.


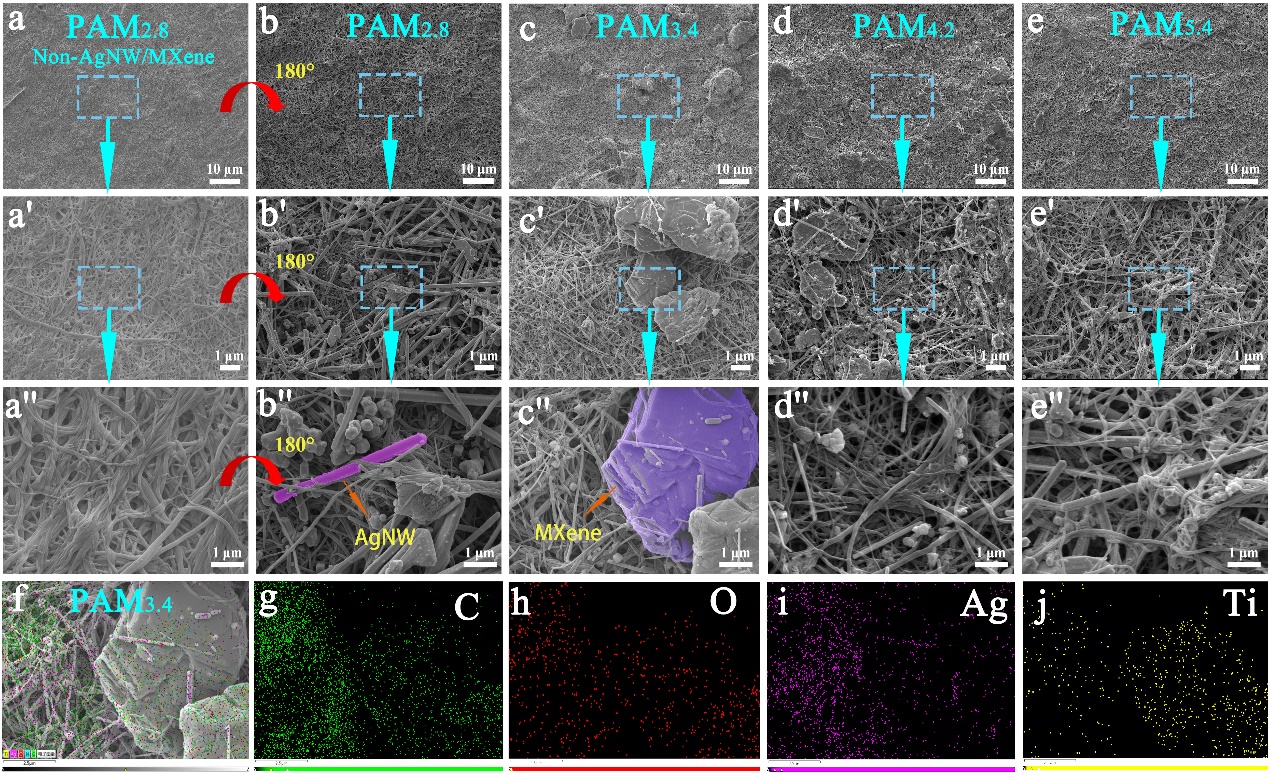


**Figure S4.** (a - e'') Surface microstructures of the PAM_x_ composite films at different magnifications. Elemental mappings of (g) C, (h) O, (i) Ag, and (j) Ti corresponding to the region shown in (f).


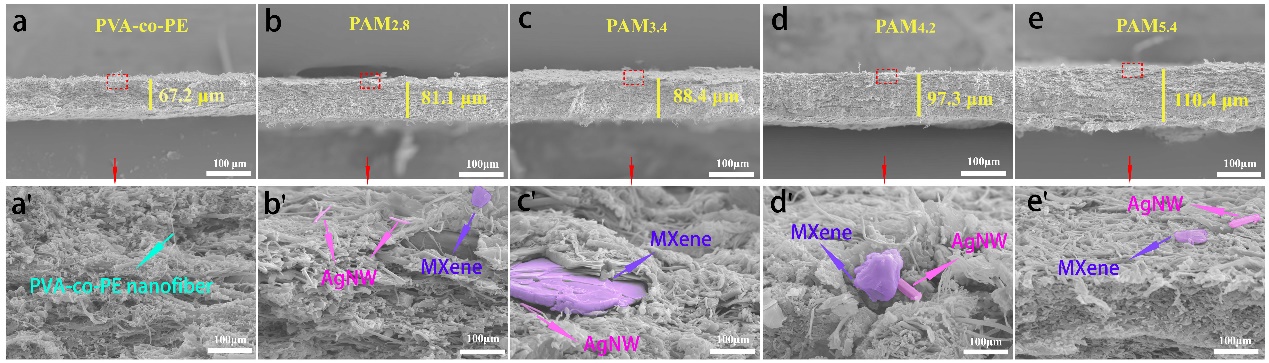


**Figure S5**. SEM images of cross-section morphologies of the PVA-co-PE, PAM_2.8_, PAM_3.4_, PAM_4.2_, PAM_5.4_ composite films.


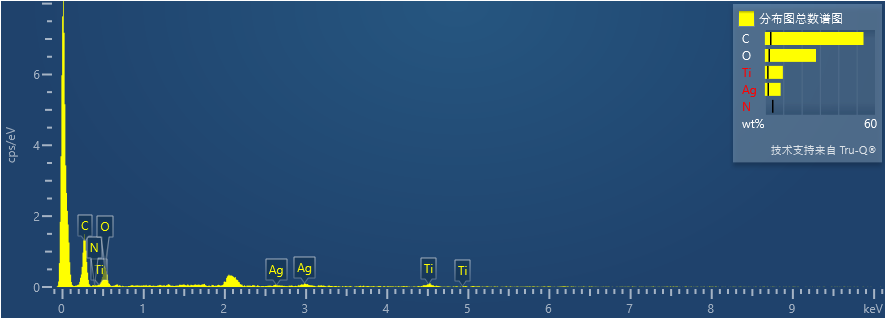


**Figure S6**. EDS spectra of the PAM_2.8_ composite film


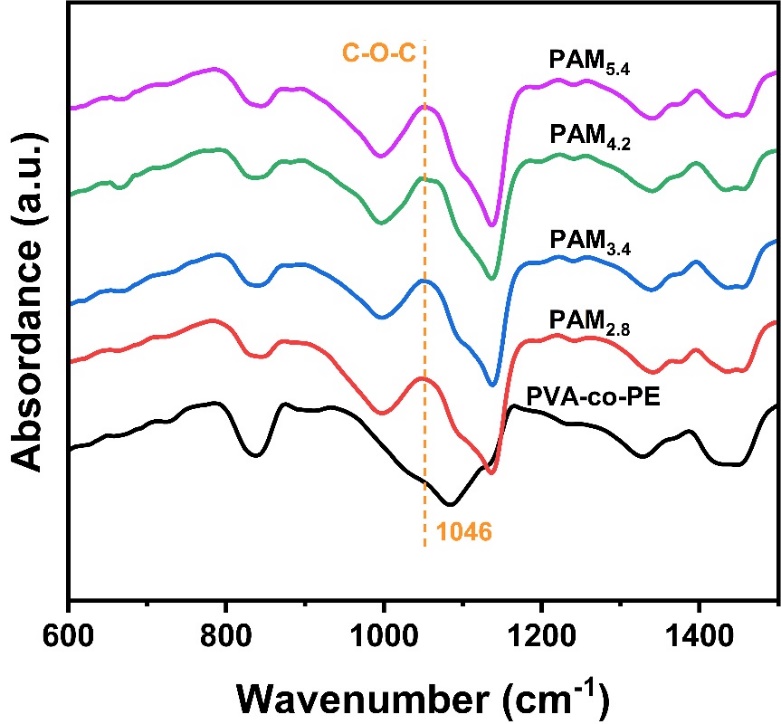


**Figure S7**. Partially enlarged FTIR spectra of different PAM_x_ composite Films.


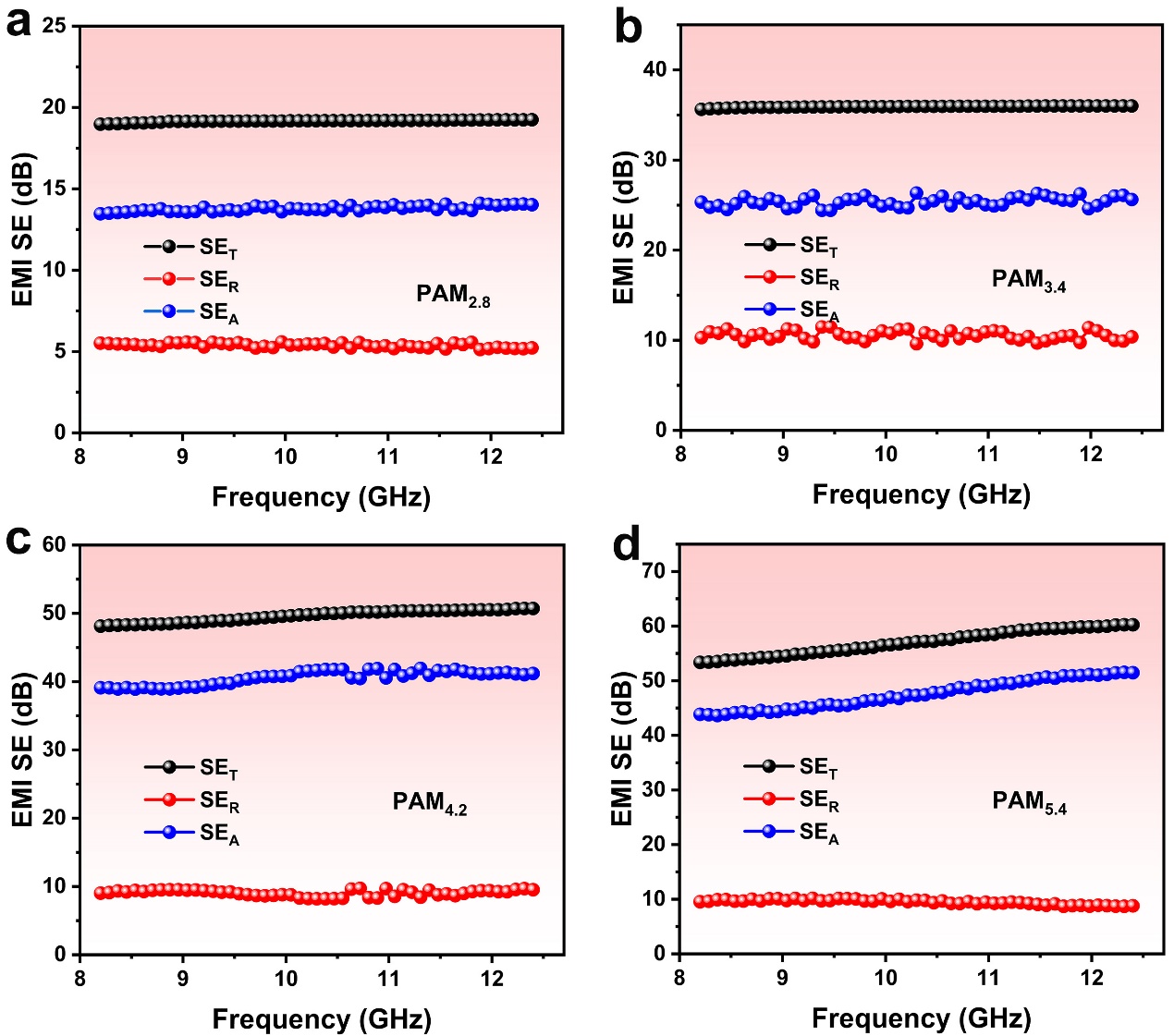


**Figure S8**. (a - d) EMI SE of different PAM_x_ heterostructured composite films.

**Table S1**. Performance comparison of PAM_x_ nanofiber composite films. with previous reported EMI shielding materials.

| **EMI shielding materials** | **Filling**  **Content (wt%)** | **Thickness**  **(μm)** | **Density**  **(g·cm^-3^)** | **EMI SE**  **(dB)** | **EMI SE/t**  **(dB·cm^−1^)** | **EMI SSE/t**  **(dB·cm^2^·g^−1^)** | **Ref.** |
| --- | --- | --- | --- | --- | --- | --- | --- |
| PVA-co-PE | 0 | 67.2 | 0.138 | / | / | / | **This work** |
| PAM_2.8_ | 2.8 | 81.6 | 0.143 | 19.2 | 2352.9 | 16453.8 |  |
| PAM_3.4_ | 3.4 | 88.4 | 0.144 | 36.0 | 4072.4 | 28280.6 |  |
| PAM_4.2_ | 4.2 | 97.3 | 0.146 | 50.7 | 5210.7 | 35689.7 |  |
| PAM_5.4_ | 5.4 | 110.4 | 0.150 | 60.2 | 5452.9 | 36352.7 |  |
| MXene/AgNWs/PVDF | 15 | 300 | 0.79 | 25.9 | 863.3 | 1091 | [2] |
| MXene/CNF film | 50 | 167 | 1.13 | 25 | 1497.0 | 884 | [3] |
| MXene/PLA | / | 150 | / | 55.4 | 3693 | / | [4] |
| MXene/CNF | 20 | 20 | / | 36.5 |  | / | [5] |
| MXene/AgNWs film | 20 | 120 | 1091 | 54.0 | 4500 | / | [6] |
| MXene/FeCo/CNF | 55 | 340 | / | 58 | 1706 | / | [7] |
| MXene/BC | 50 | 116 | / | 43.7 | 3767 | / | [8] |
| MXene/Ni/PVDF | 20 | 100 | / | 19.5 | 1950 | / | [9] |
| MXene-WPU-Co/C | 10 | 200 | / | 31 | / | / | [10] |
| PANI/MXene | / | 300 | 0.06 | 36.3 | 1210 | / | [11] |
| Ti_3_C_2_T*_x_*/Wax | / | 800 | 2.03 | 70 | 875 | 431 | [12] |
| Ti_3_C_2_T*_x_*/PVA | 0.15 | 5000 | 0.011 | 28 | 56 | 5136 | [13] |
| MXene | 100 | 11 | 0.001 | 68 | 28.4 | 25863 | [14] |
| Graphene oxide/Fe_3_O_4_ paper | 50 | 300 | 0.78 | 24 | 800 | 1025.6 | [15] |
| LPGF | 100 | 200 | 0.075 | 43.8 | 2190 | 29178 | [16] |
| PPGF | 100 | 200 | 0.078 | 22 | 1100.0 | 14103 | [16] |
| Ni@graphene/PVDF | 20 | 700 | / | 51.4 | 734 | / | [17] |
| RGO/PS | 3.47 | 2500 | 0.25 | 45.1 | 41.9 | 167.5 | [18] |
| RGO/PDMS | 0.36 | 1000 | 0.1 | 20 | 333.3 | 3333 | [19] |
| RGO/WPU | 5 | 1000 | / | 34 | 33.8 | 338 | [20] |
| RGO-γ-Fe2O3 | 2.3 | 360 | / | 20.3 | 15 | 416.7 | [21] |
| MWCNT/GR/PPy | / | 249 | / | 59.6 | / | / | [22] |
| RGO/PMMA | 1.8 | 4000 | 0.79 | 19 | 47.4 | 60 | [23] |
| Carbon nanotube/AgNWs/cellulose paper | 6.07 | 160 | 0.51 | 23.8 | 1487.5 | 2916.7 | [24] |
| MWCNT/WPU | 76.2 | 100 | 0.04 | 21.1 | 211 | 5140 | [25] |
| MWCNT/WPU | 76 | 320 | 0.45 | 49 | 1531 | 3408 | [26] |
| CNF/MXene/FeCo@rGO | 20 | 192 | / | 45.2 | / | / | [27] |
| MWCNT/WPU | 7.2 | 4500 | 0.126 | 50 | 111.1 | 881.8 | [25] |
| MWCNT/PLLA | 1.47 | 2500 | 0.299 | 23 | 92 | 306.7 | [28] |
| PANI/MWCNT/MoS_2_ | 5.0 | 280 | / | 48.9 | / | / | [29] |
| CNWs@G | 4.6 | 1600 | 0.159 | 36 | 370.7 | 2317 | [30] |
| CNT/sponge | / | 2400 | 0.02 | 22 | 91.7 | 4583 | [31] |
| MWCNT/CNF | / | 150 | 0.77 | 46.4 | 3093.3 | 4017.3 | [32] |
| AgNWs/calcium alginate/PU film | 10 | 330 | 0.174 | 31.3 | 948.5 | 5451.1 | [33] |
| AgNWs/PI | 4.5 | 5000 | 0.029 | 35 | 70 | 2416 | [34] |
| AgNWs/PANI | 14 | 13 | / | 48 | 36923 | / | [35] |
| AgNWs/cellulose papers | 0.53 | 160 | 0.53 | 48.6 | 3038 | 5585 | [36] |
| AgNWs/PS | / | 800 | / | 33 | / | / | [34] |
| AgNWs/Epoxy | / | 13 | / | 50 | / | / | [35] |

**References**

1. Tao, D.; Wen, X.; Yang, C.; Yan, K.; Li, Z.; Wang, W.; Wang, D., *Nano-Micro Letters* **2024,** *16* (1), 236. DOI 10.1007/s40820-024-01444-y.

2. Cheng, H.; Pan, Y.; Chen, Q.; Che, R.; Zheng, G.; Liu, C.; Shen, C.; Liu, X., *Advanced Composites and Hybrid Materials* **2021,** *4* (3), 505-513. DOI 10.1007/s42114-021-00224-1.

3. Cao, W.-T.; Chen, F.-F.; Zhu, Y.-J.; Zhang, Y.-G.; Jiang, Y.-Y.; Ma, M.-G.; Chen, F., *ACS Nano* **2018,** *12* (5), 4583-4593. DOI 10.1021/acsnano.8b00997.

4. Du, Z.; Chen, K.; Zhang, Y.; Wang, Y.; He, P.; Mi, H.-Y.; Wang, Y.; Liu, C.; Shen, C., *Composites Communications* **2021,** *26*, 100770. DOI <https://doi.org/10.1016/j.coco.2021.100770>.

5. Feng, S.; Zhan, Z.; Yi, Y.; Zhou, Z.; Lu, C., *Composites Part A: Applied Science and Manufacturing* **2022,** *157*. DOI 10.1016/j.compositesa.2022.106907.

6. Liu, L.-X.; Chen, W.; Zhang, H.-B.; Wang, Q.-W.; Guan, F.; Yu, Z.-Z., *Advanced Functional Materials* **2019,** *29* (44), 1905197. DOI <https://doi.org/10.1002/adfm.201905197>.

7. Ma, M.; Tao, W.; Liao, X.; Chen, S.; Shi, Y.; He, H.; Wang, X., *Chemical Engineering Journal* **2023,** *452*, 139471. DOI <https://doi.org/10.1016/j.cej.2022.139471>.

8. Liu, H.; Cui, Z.; Luo, L.; Liao, Q.; Xiong, R.; Xu, C.; Wen, C.; Sa, B., *Chemical Engineering Journal* **2023,** *454*, 140288. DOI <https://doi.org/10.1016/j.cej.2022.140288>.

9. Wang, S.-J.; Li, D.-S.; Jiang, L., *Advanced Materials Interfaces* **2019,** *6* (19), 1900961. DOI <https://doi.org/10.1002/admi.201900961>.

10. Zhang, G.; Du, M.; Tan, Z.; Zhao, H.; Yi, L.; Ji, X.; Zhao, L., *Materials Today Communications* **2023,** *37*. DOI 10.1016/j.mtcomm.2023.107536.

11. Song, S.; Xu, G.; Wang, B.; Liu, D.; Ren, Z.; Wang, C.; Zhao, J.; Zhang, L.; Li, Y., *ACS Applied Materials & Interfaces* **2022,** *14* (46), 52379-52389. DOI 10.1021/acsami.2c14841.

12. Li, X.; Yin, X.; Liang, S.; Li, M.; Cheng, L.; Zhang, L., *Carbon* **2019,** *146*, 210-217. DOI <https://doi.org/10.1016/j.carbon.2019.02.003>.

13. Xu, H.; Yin, X.; Li, X.; Li, M.; Liang, S.; Zhang, L.; Cheng, L., *ACS Applied Materials & Interfaces* **2019,** *11* (10), 10198-10207. DOI 10.1021/acsami.8b21671.

14. Shahzad, F.; Alhabeb, M.; Hatter, C. B.; Anasori, B.; Man Hong, S.; Koo, C. M.; Gogotsi, Y., *Science* **2016,** *353* (6304), 1137-1140. DOI doi:10.1126/science.aag2421.

15. Song, W.-L.; Guan, X.-T.; Fan, L.-Z.; Cao, W.-Q.; Wang, C.-Y.; Zhao, Q.-L.; Cao, M.-S., *Journal of Materials Chemistry A* **2015,** *3* (5), 2097-2107. DOI 10.1039/C4TA05939E.

16. Lai, D.; Chen, X.; Wang, G.; Xu, X.; Wang, Y., *Journal of Materials Chemistry C* **2020,** *8* (26), 8904-8916. DOI 10.1039/D0TC01346C.

17. Liang, L.; Xu, P.; Wang, Y.; Shang, Y.; Ma, J.; Su, F.; Feng, Y.; He, C.; Wang, Y.; Liu, C., *Chemical Engineering Journal* **2020,** *395*, 125209. DOI <https://doi.org/10.1016/j.cej.2020.125209>.

18. Yan, D. X.; Pang, H.; Li, B.; Vajtai, R.; Xu, L.; Ren, P. G.; Wang, J. H.; Li, Z. M., *Advanced Functional Materials* **2015,** *25* (4), 559-566.

19. Chen, Z.; Xu, C.; Ma, C.; Ren, W.; Cheng, H. M., *Advanced materials* **2013,** *25* (9), 1296-1300.

20. Hsiao, S.-T.; Ma, C.-C. M.; Liao, W.-H.; Wang, Y.-S.; Li, S.-M.; Huang, Y.-C.; Yang, R.-B.; Liang, W.-F., *ACS Applied Materials & Interfaces* **2014,** *6* (13), 10667-10678. DOI 10.1021/am502412q.

21. Yuan, B.; Bao, C.; Qian, X.; Song, L.; Tai, Q.; Liew, K. M.; Hu, Y., *Carbon* **2014,** *75*, 178-189.

22. Xie, Z.; Chen, H.; Hu, S.; Zhao, H.; Chen, W.; Jiang, D., *Polymer Composites* **2023,** *44* (7), 3798-3807. DOI 10.1002/pc.27357.

23. Zhang, H.-B.; Yan, Q.; Zheng, W.-G.; He, Z.; Yu, Z.-Z., *ACS Applied Materials & Interfaces* **2011,** *3* (3), 918-924. DOI 10.1021/am200021v.

24. Choi, H. Y.; Lee, T.-W.; Lee, S.-E.; Lim, J.; Jeong, Y. G., *Composites Science and Technology* **2017,** *150*, 45-53. DOI <https://doi.org/10.1016/j.compscitech.2017.07.008>.

25. Zeng, Z.; Jin, H.; Chen, M.; Li, W.; Zhou, L.; Zhang, Z., *Advanced Functional Materials* **2016,** *26* (2), 303-310. DOI <https://doi.org/10.1002/adfm.201503579>.

26. Zeng, Z.; Chen, M.; Jin, H.; Li, W.; Xue, X.; Zhou, L.; Pei, Y.; Zhang, H.; Zhang, Z., *Carbon* **2016,** *96*, 768-777. DOI <https://doi.org/10.1016/j.carbon.2015.10.004>.

27. Ma, M.; Shao, W.; Chu, Q.; Tao, W.; Chen, S.; Shi, Y.; He, H.; Zhu, Y.; Wang, X., *Journal of Materials Chemistry A* **2024,** *12* (3), 1617-1628. DOI 10.1039/d3ta06467k.

28. Kuang, T.; Chang, L.; Chen, F.; Sheng, Y.; Fu, D.; Peng, X., *Carbon* **2016,** *105*, 305-313.

29. Dhanasekaran, A.; Dhanaraj, K.; Venugopal, V., *ACS Applied Nano Materials* **2024,** *7* (22), 25983-25994. DOI 10.1021/acsanm.4c05214.

30. Kong, L.; Yin, X.; Han, M.; Yuan, X.; Hou, Z.; Ye, F.; Zhang, L.; Cheng, L.; Xu, Z.; Huang, J., *Carbon* **2017,** *111*, 94-102. DOI <https://doi.org/10.1016/j.carbon.2016.09.066>.

31. Crespo, M.; González, M.; Elías, A. L.; Pulickal Rajukumar, L.; Baselga, J.; Terrones, M.; Pozuelo, J., *physica status solidi (RRL) – Rapid Research Letters* **2014,** *8* (8), 698-704. DOI <https://doi.org/10.1002/pssr.201409151>.

32. Zhang, H.; Sun, X.; Heng, Z.; Chen, Y.; Zou, H.; Liang, M., *Industrial & Engineering Chemistry Research* **2018,** *57* (50), 17152-17160. DOI 10.1021/acs.iecr.8b04573.

33. Jia, L.-C.; Yan, D.-X.; Liu, X.; Ma, R.; Wu, H.-Y.; Li, Z.-M., *ACS Applied Materials & Interfaces* **2018,** *10* (14), 11941-11949. DOI 10.1021/acsami.8b00492.

34. Ma, J.; Wang, K.; Zhan, M., *RSC Advances* **2015,** *5* (80), 65283-65296. DOI 10.1039/C5RA09507G.

35. Fang, F.; Li, Y.-Q.; Xiao, H.-M.; Hu, N.; Fu, S.-Y., *Journal of Materials Chemistry C* **2016,** *4* (19), 4193-4203. DOI 10.1039/C5TC04406E.

36. Lee, T.-W.; Lee, S.-E.; Jeong, Y. G., *ACS Applied Materials & Interfaces* **2016,** *8* (20), 13123-13132. DOI 10.1021/acsami.6b02218.
